# Supplementary material for: Early impacts of the COVID-19 pandemic on mental health care and on people with mental health conditions: framework synthesis of international experiences and responses
Source: Soc Psychiatry Psychiatr Epidemiol. 2020 Aug 17;56(1):13–24. doi: 10.1007/s00127-020-01924-7 (PMC7429938; doi:10.1007/s00127-020-01924-7)
Supplement: Supplementary file 1 — Supplementary file1 (DOCX 239 kb) [file 127_2020_1924_MOESM1_ESM.docx]

**Supplementary Report; First Reports regarding impacts of the COVID-19 pandemic on mental health care and people with mental health conditions: an international documents analysis**

**Contents**

[**Section 1 - Results** 2](#_Toc41810893)

[**Table 1.** *Coding Framework* 2](#_Toc41810894)

[**Table 2a.** *Impacts on mental health of people with pre-existing conditions* 6](#_Toc41810895)

[**Table 2b.** *Surveys on the impact of the covid-19 pandemic on the mental health of people living with mental illness* 11](#_Toc41810896)

[**Table 3.** *Loneliness, social isolation and loss of routines and activities* 13](#_Toc41810897)

[**Table 4.***Access to essential services & resources: reports on individual experiences* 16](#_Toc41810898)

[**Table 5.***Family and social adversities and safety concerns* 19](#_Toc41810899)

[**Table 6.** *COVID infection risks and risks of severe illness for people living with mental health problems* 21](#_Toc41810900)

[**Table 7.** *Positive experiences of life in the pandemic period* 24](#_Toc41810901)

[**Table 8a.***Strategies people with lived experience employ to manage life in pandemic period* 26](#_Toc41810902)

[**Table 8b.** *Peer support* 29](#_Toc41810903)

[**Table 9.** *How has service activity changed? Reports on changes in the early part of the pandemic* 32](#_Toc41810904)

[**Table 10.** *Challenges in inpatient and residential settings during the pandemic* 34](#_Toc41810905)

[**Table 11.** *Service adaptations and innovations in inpatient and residential settings* 37](#_Toc41810906)

[**Table 12.** *Challenges in community settings* 39](#_Toc41810907)

[**Table 13.** *Service adaptation and innovations in community settings* 41](#_Toc41810908)

[**Table 14.** *Ethical challenges and challenges to values:* *Inequalities, denial of treatment or provision of inadequate treatment, challenges associated with compulsory admission* 44](#_Toc41810909)

[**Table 15.** *What do people anticipate, hope for, or fear in the future?* 46](#_Toc41810910)

[**Section 2 - Search Strategy** 49](#_Toc41810911)

[**Section 3 – Online Data Collection Form** 62](#_Toc41810912)

# **Section 1 - Results**

## **Table 1.** *Coding Framework*

|  |  |  |
| --- | --- | --- |
| **Direct impacts on mental health problems** | Impacts on mental health of people with pre-existing conditions | Negative impact on mental health |
|  |  | Depression and anxiety |
|  |  | Obsessive compulsive disorder (OCD) |
|  |  | Eating disorders |
|  |  | Psychosis |
|  |  | Substance misuse disorders |
|  |  | Bipolar disorder |
|  |  | Post-traumatic stress disorder (PTSD) |
|  |  | Self-harm and “personality disorder” |
|  |  | Children and adolescents with mental health problems |
|  |  | Older people with mental health conditions |
|  |  | Disrupted health-relevant behaviours in the upheaval of the pandemic |
|  |  | Changes in substance use among people with mental health problems |
|  | Experiences of people with mental health problems | Loneliness and isolation impact on mental health |
|  |  | Disruption to routines and activities |
|  |  | Impact of lack of seeing family/friends/social contact |
|  |  | Impact of closed mental health facilities, therapeutic activities and support groups |
|  |  | Social impact of lack of visits and lack of social contact in inpatient/residential settings |
|  |  | Confinement in inpatient facilities and lack of activities |
|  |  | Adverse living conditions (small, chaotic, family tension, domestic violence) |
|  | Access to essential services & resources: reports on individual experiences | Lack of access to mental health and other types of service |
|  |  | "We are not all in this together": COVID-19 magnifying existing inequalities and creating new ones |
|  |  | Insufficiency of remote services |
|  |  | Medication access |
|  |  | Hesitation to seek help |
|  |  | Pressures on already stretched services |
|  |  | Increased experiences of economic hardship and lack of basic resources |
|  | Family and social adversities and safety concerns | Strain on family and carers |
|  |  | Increased risk of violence |
|  |  | Concerns about family relationships |
|  |  | Support for families |
|  | COVID infection risks and risks of severe illness for people with mental health problems | Challenges for people with mental health problems in following infection control measures |
|  |  | Experience of outbreaks |
|  |  | Increased health anxiety related to COVID-19 |
|  |  | Increased risk of being infected, or of infection being severe |
|  |  | Interactions between COVID-19 and multiple needs, disadvantages and competing mental and physical health problems |
|  |  | Stigma as a barrier to treatment |
|  | Positive experiences of life in the pandemic period | Coping well with lockdown measures |
|  |  | Shared experiences and solidarity |
|  |  | Improvement in mental health |
|  |  | Improvements to support |
|  |  | More time |
| **Individual coping strategies in the times of the pandemic** | Strategies people with lived experience employ to manage life in pandemic period | Maintaining health and wellbeing |
|  |  | Social contact |
|  |  | Structure & routine |
|  |  | Limiting information |
|  |  | Self-management tools |
|  |  | Carer strategies |
|  | Peer support | Practical and emotional support |
|  |  | Creative digital and online approaches |
|  |  | Communicating and connecting |
|  |  | Family and friends |
|  |  | Helping and contributing |
|  |  | Sharing stories and experiences |
|  |  | Community support |
| **Service impacts** | How has service activity changed? Reports on changes in the early part of the pandemic | Helpline |
|  |  | Hospital |
|  |  | Digital |
|  |  | Community |
|  |  | Accident & Emergency |
|  |  | Medication |
| **Service challenges and adaptations in inpatient and residential settings** | Challenges in inpatient and residential settings during the pandemic | Infection control in psychiatric units/residential settings |
|  |  | Inpatient restrictions |
|  |  | De-prioritisation |
|  |  | Staffing and expertise available |
|  | Service adaptations and innovations in inpatient and residential settings | COVID-19 specific units |
|  |  | Technology |
|  |  | Links with community |
|  |  | Initiatives to maintain physical distancing on wards |
|  |  | Services maintained |
|  |  | Introducing Personal Protective Equipment |
| **Service challenges and adaptations in community settings** | Challenges in community settings | Disruption to services |
|  |  | Workforces issues |
|  |  | Inadequate PPE |
|  |  | Impediments to face to face treatment |
|  |  | Challenges with remote care |
|  |  | Workforce challenges |
|  |  | Financial challenges |
|  |  | Lack of access to tests |
|  | Service adaptation and innovations in community settings | The shift to telehealth |
|  |  | Increasing access |
|  |  | Reorganisation of services |
|  |  | Recognising mental health services as essential |
| **Ethical challenges** | Ethical challenges and challenges to values: Inequalities, denial of treatment or provision of inadequate treatment, challenges associated with compulsory admission | Inequitable access to physical health care |
|  |  | Less ethical care, especially in inpatient units |
|  |  | Legislative changes curtailing rights |
|  |  | Difficulties accessing advocacy/legal support |
| **Expectations and concerns for future** | What do people anticipate, hope for, or fear in the future? | Increased demand and new strategies |
|  |  | Socio-economic challenges |
|  |  | Use of technology |
|  |  | Challenges for people with mental health problems |
|  |  | Positive considerations and hopes |
|  |  | New outbreak and virus infection |

**Direct impacts on mental health problems**

## **Table 2a.** *Impacts on mental health of people with pre-existing conditions*

**Total unique sources:**715

**Theme summary:**

Many sources, including clinicians and people living with mental health problems as well as journalists (often based on interviews with people with mental health conditions) described negative impacts on mental health in people with pre-existing mental health conditions as well as exacerbation of symptoms. The major routes to such impacts tended to be increased anxiety and fear of illness and death directly related to COVID-19, impacts of “lockdowns”  and social distancing policies, especially through social isolation, interactions between symptoms of mental health problems and current public events and concerns, effects of loss of support from health and other services, and effects of increased social adversities, such as from domestic violence, family conflict or loss of employment. Some accounts were based on direct experience, whereas other authors seemed rather to be reporting predictions or expectations. People with depression and anxiety, obsessive compulsive disorder and eating disorders are prominent in writing about the early effects of the pandemic, with immediate impacts reported. Large variations in individual responses are often described, especially by clinicians regarding the range of patients whom they see, with some deteriorating but others coping relatively well with the early phases of the pandemic, drawing on reserves of resilience in adversity.

| *Theme* | *Number of sources* | *Countries* | *Author types^a^* | *Source type^b^* | *Content* |
| --- | --- | --- | --- | --- | --- |
| Negative impact on mental health | 426 | UK (108), USA (78), Germany (52), France (42), Italy (42), International (38), Spain (19), China (11), Switzerland (7), Australia (6), Canada (4), Belgium (3), India (3), Austria (2), Costa Rica (2), Colombia (1), Dominican Republic (1), Ghana (1), Indonesia (1), Ireland (1), Mexico (1), New Zealand (1), Puerto Rico (1), Qatar (1) | Journalist (227), Clinician (79), Person with relevant lived experience (61), Policy, professional and charity sector (49), Scientist (46), Unknown (15) | Media article (222), Organisational website (97), Specialist health/social care press (48), Journal article (29), Video/Webinar/Podcast (16), Policy body (9), Blog (5) | Many sources described negative impacts on mental health in people with pre-existing mental health conditions and exacerbation of symptoms. The major routes to such impacts tended to be increased anxiety, and fear of illness and death directly related to COVID-19, Impacts of “lockdowns”  and social distancing policies, especially of social isolation, interactions between symptoms of mental health problems and current public events and concerns, impacts of loss of support from health and other services, and effects of increased social adversities, such as from domestic violence, family conflict or loss of employment. Some accounts (see following sub-themes) described impacts on specific mental health conditions. Others, often vaguer in their descriptions of pathways and impacts, simply described an overall negative impact.    Fourteen sources related to surveys conducted by scientists or voluntary sector bodies (sometimes more than one source discussing the same survey) these are outlined in the *Findings* section of the paper and in Table 1 above. Of the others, 113 sources appeared more to describe an assumption or prediction of an impact on mental health, rather than presenting any evidence or narratives to support their claims. These included several articles predicting a marked rise in suicide, a prediction that an international collaboration argues should not be accepted as an inevitability, but mitigated through rapid development of suicide prevention strategies (Gunnell et al., 2020)^c^. Sixty-nine sources reported personal experiences of people with pre-existing mental health conditions, often with detailed accounts of the impact of the pandemic period on their own symptoms and daily challenges. Two hundred and twenty-nine sources were from authors describing their observations of other people or the narratives recounted by others, for example clinicians’ observations of their patients or journalists’ narratives regarding people they had interviewed.    Of note, many of these accounts, especially from clinicians, made it clear that they had observed worsening mental health in some, but certainly not all, of the people discussed. Many noted great variations in responses to the pandemic, with some people with mental health conditions resilient in adversity, deploying coping skills learnt over many years, and some even describing some positive effects of the lock-down period (see Table 7). |
| Depression and anxiety | 115 | Germany (27), USA (24), UK (19), France (11), International (9), Spain (6), Switzerland (6), Italy (5), Canada (3), China (3), India (1), Qatar (1) | Journalist (73), Person with relevant lived experience (16), Clinician (14), Scientist (8), Policy, professional and charity sector (7), Unknown (5) | Media article (78), Organisational website (18), Specialist health and social care press (9), Video/Webinar/Podcast (6), Journal article (4) | Accounts of exacerbation of depression and anxiety were frequent. Identified pathways to worsened depression and/or anxiety included the shutting down in the “lockdown” period of routines, activities and resources (such as therapy) that usually maintain good mental health (26 sources); isolation from family and friends and other supportive contacts (19 sources) (some noted that the social withdrawal required in the pandemic is the opposite of the behaviour recommended as therapeutic in depression); direct fears of infection, severe illness and possible death, sometimes interacting with pre-existing health anxiety (18 sources); and impacts of the social environment during the pandemic, such as constant negative news coverage and panic-buying (22 sources). |
| Obsessive compulsive disorder  (OCD) | 81 | USA (25), UK (17), Spain (13), Germany (9), International (4), France (3), China (2), India (2), Australia (1), Canada (1), Dominican Republic (1), Mexico (1), Puerto Rico (1), Switzerland (1) | Journalist (48), Person with relevant lived experience (20), Clinician (14), Policy, professional and charity sector (5), Scientist (2), Unknown (1) | Media article (53), Organisational website (17), Specialist health and social care press (6), Video/Webinar/Podcast (3), Blog (1), Journal article (1) | We found many accounts of relapse or fear of relapse in OCD, including worsening of catastrophic thinking and of anxiety symptoms, and increased frequency of compulsive behaviours (47 sources), especially for people whose symptoms related to fear of contamination. Guidance such as on frequent handwashing is a potential exacerbating factor for obsessions and/or compulsions related to hygiene, and conflicts with response prevention techniques used in treatment (31 sources). Development of new anxieties focused on the virus was also reported (23 sources), with some describing the pandemic as the realisation of their worst fears. However, accounts also indicated that deterioration is not inevitable, with some people with OCD successfully mobilising coping strategies learnt over the years. |
| Eating disorders | 46 | UK (14), International (10) USA (9), France (6), Germany (2), Australia (1), Canada (1), New Zealand (1), Switzerland (1) | Journalist (21), Person with relevant lived experience (10), Policy, professional and charity sector (7), Clinician (6), Scientist (3), Unknown (1) | Media article (27), Organisational website (14), Journal article (2), Specialist health and social care press (1), Video/Webinar/Podcast (1) | Multiple sources reported the often-rapid onset of negative effects of the pandemic and attendant lockdown on people with eating disorders, exacerbating current symptoms or increasing risk of relapse. Sources described a loss of control through disruption of usual routines, both those associated with eating, and the social routines and daily activities generally found helpful in maintaining healthy eating patterns (33 sources). Direct effects of disruptions of food supplies and the societal preoccupation with food were also described (16 sources): lack of access to “safe” foods, temptations to binge or feelings of guilt associated with stockpiling of food, and pressures associated with the much greater prominence of food in media and interpersonal discussions. Disruption of usual exercise routines, with gym closures and lack of access to outside exercise, intensified fears about gaining weight, or feelings of guilt for some (14 sources). Other reported risk factors for deterioration were withdrawal of treatment programmes (7 sources) and intensified family conflict (4 sources). |
| Psychosis | 36 | Italy (9), UK (7), France (6), Germany (4), USA (4), China (2), International (2), Spain (1), Switzerland (1) | Journalist (19), Clinician (9), Policy, professional and charity sector (3), Scientist (3), Unknown (3), Person with relevant lived experience (1) | Media article (18), Organisational website (11), Specialist health and social care press (4), Journal article (3) | Thirty-six sources, many from clinicians, reported on actual or anticipated effects on people with psychosis, including those with a diagnosis of schizophrenia. Many of these described worsening of psychotic symptoms and/or increased anxiety. Social triggers were often identified for these, including isolation, loss of usual routines and activities developed to maintain mental health, loss of important social contacts, including in mental health settings, and loss of visits for people in hospitals or residential settings. Considerable variations were described, with some people with psychosis long accustomed to adversity and isolation and responding stoically. Direct interactions between the pandemic and delusional ideas were described by eight sources, including development of COVID-19 related delusions and impacts on people with delusional ideas congruent with the pandemic crisis. |
| Substance misuse disorders | 20 | UK (7), France (5), International (2), Italy (2), USA (2), Australia (1), Germany (1) | Journalist (7), Policy, professional and charity sector (6), Clinician (5), Scientist (5), Person with relevant lived experience (1), Unknown (1) | Media article (7), Policy body (4), Organisational website (3), Specialist health and social care press (2), Video/Webinar/Podcast (2), Blog (1), Journal article (1) | The stresses of the pandemic, including anxiety about the future, boredom, loss of usual activities and routines and increased time alone were reported as contributing to relapse or increased consumption among people with substance misuse disorders (12 sources). There were also specific concerns regarding lack of access to treatment (7 sources): facilities for supported withdrawal from alcohol appeared to have been widely withdrawn, many group programmes supporting abstinence had been suspended, and access to opioid maintenance programmes was a further reported difficulty, with concerns regarding possible overdose for people given larger than usual maintenance opioid supplies. Impacts of inadvertent withdrawal from alcohol or street drugs were a further concern. |
| Bipolar disorder | 19 | France (4), Spain (4), UK (4), International (3), UK (2), China (1), Indonesia (1) | Journalist (9), Person with relevant lived experience (8), Clinician (2), Scientist (2), Policy, professional and charity sector (1 | Media article (9), Organisational website (5), Video/Webinar/Podcast (3), Blog (1), Specialist health and social care press (1) | Some sources described relapses in bipolar disorder, with several risk factors identified for both depressive and manic mood swings, some resembling those described above under Depression and Anxiety. Concerns more specifically related to bipolar included the importance of routine and calming self-management activities in remaining stable in bipolar disorder, and the disruption to this associated with the pandemic. |
| Post-traumatic stress disorder  (PTSD) | 7 | Germany (3), USA (2), France (1), Spain (1) | Journalist (6), Scientist (2), Clinician (1) | Media article (6), Journal article (1) | A few sources were retrieved on potential impacts of the pandemic on people with pre-existing PTSD (many discussing potential new cases of PTSD were excluded). The general stress of the crisis and the loss of social support was predicted to worsen symptoms and increase relapse risk. Healthcare professionals report concerns that the crisis, and traumatic events such as bereavements during this time, may result in re-traumatisation for people with PTSD, and that long periods in confined environments may further exacerbate symptoms for some. |
| Self-harm and “personality disorder” | 9 | UK (6), France (1), Italy (1), USA (1) | Person with relevant lived experience (5), Journalist (2), Clinician (1), Policy, professional and charity sector (1), Scientist (1) | Media article (3), Organisational website (3), Specialist health and social care press (3) | Nine sources discussed impact on people with repeated impulses to harm themselves and/or a diagnosis of “borderline” or “emotionally unstable personality disorder”. Potential triggers for deterioration included loss of the routines and social contacts that people have developed to maintain stability and avoid self-harm, lack of access to and feelings of abandonment by usual formal and informal sources of support, and difficulty managing enforced solitude. Difficulty accessing or reluctance to access emergency departments for help needed following self-harm was a further concern. |
| Children and adolescents with mental health problems | 8 | France (3), Italy (2), UK (2), International (1) | Clinician (3), Journalist (3), Policy, professional and charity sector (1), Scientist (1), Unknown 1) | Media article (3), Organisational website (2), Specialist health and social care press (2), Journal article (1) | We found eight sources on impact on children and adolescents with mental health problems, in which a very high level of concern tended to be expressed regarding the impact of social consequences of the pandemic. In particular, the loss of social contacts, structure, and support at school were seen as a major problem for many children and adolescents with mental health problems. Pressures on families now receiving little support in looking after children with mental health or developmental difficulties at home, especially those with challenging behaviours, were seen as severe. |
| Older people with mental health conditions | 3 | China (2), UK (1) | Scientist (2), Unknown (1) | Journal article (2), Organisational website (1) | Three sources yielded specific discussions of older people with mental health problems (dementia was not included in our search). Specific difficulties identified included greater risk of severe COVID-19 infection, resulting in greater impact on mental health problems and greater need to avoid face to face contact or hospital admission; consequences of cognitive impairment for following infection control guidance and understanding the current situation; and greater risk of loss of contact with services due to a combination of face to face contact being less safe and older people being less accessible by remote and digital methods. |
| Disrupted health-relevant behaviours in the upheaval of the pandemic | 23 | Germany (4), UK (4), USA (3), France (3), Australia (1), China (1), New Zealand (1) | Journalist (8), Clinician (8), Person with relevant lived experience (5), Scientist (3), Policy, professional and charity sector (1), Unknown (1) | Media article (11), Organisational website (4), Specialist health and social care press (3), Journal article (2), Blog (1), Policy body (1), Video/Webinar/Podcast (1) | A theme spanning health conditions was disrupted health behaviours in a context of restrictions and stress. These included changes to diet, exercise, or impaired/excessive sleep, with impacts on a range of mental health conditions. Some pieces discussed a negative cycle, such as impaired sleep leading to negative mood leading to impaired sleep. |
| Changes in substance use among people with mental health problems | 22 | UK (7), China (6), International (2), USA (2), France (1), Germany (1), India (1), Italy (1), Spain (1) | Many of the sources were identified using web search engines. Search results from these are influenced by factors such as time of day and IP address, limiting replicability and comprehensiveness. Our English-language search strategy was extensive than for other languages, especially because English-speaking experts contributed additional sources. | Media article (7), Organisational website (5), Blog (3), Journal article (3), Policy body (2), Specialist health and social care press (1), Video/Webinar/Podcast (1) | A further theme relevant across mental health conditions was a change in substance use or other potentially addictive behaviours, such as gambling, in response to the pandemic, accompanied by risk of exacerbating mental health conditions. |

1. Source types separate articles in media outlets for the general public, from those in publications with a specialist focus on health, social care or related areas.
2. Authors are in some case double counted where authors fitted more than one of these categories.
3. *Gunnell, D., Appleby, L., Arensman, E., Hawton, K., John, A., Kapur, N., ... & Chan, L. F. (2020). Suicide risk and prevention during the COVID-19 pandemic. The Lancet Psychiatry, 7(6), 468-471.*

## **Table 2b -** *Surveys on the impact of the covid-19 pandemic on the mental health of people living with mental illness*

| **Survey** | **Methodology** | **Key results** |
| --- | --- | --- |
| YoungMinds^a^ | Survey of 2,111 young people with a history of mental health problems.  Carried out between 20^th^ March and 25^th^ of March 2020 | 83% of respondents felt that the coronavirus pandemic and subsequent public health responses had caused a negative impact on their mental health, 32% felt that it had made their mental health much worse  Respondents reported increased levels of anxiety and panic attacks, sleep problems and thoughts of self-harming. |
| Rethink^b^ | Ongoing online survey with close to 800 responses from people living with mental health problems as of 27.04.2020 | 80% of respondents reported that the coronavirus pandemic and public health measures had caused a negative impact on their mental health, 28% felt that it had made their mental health much worse  Specific reasons for worsening mental health included not being able to see friends and family (reported by 69%), due to receiving less support from mental health services (reported by 47%), and the increased precarity of their employment situation (reported by 29%). |
| The Academy of Medical Sciences/MQ^c^ | Online survey of 2,198 stakeholders including people with lived experience of mental health problems, healthcare professionals, researchers and the general public with an interest in mental health.  Carried out between 25^th^ March and 27^th^ of March 2020 | Key concerns about the impact of the COVID-19 pandemic on mental health:   - Increased anxiety brought on by the pandemic - Loneliness and isolation due to social distancing - An increased risk of becoming mentally unwell, and worsening of mental health problems - Issues in accessing mental health services - Family and relationship difficulties |
| Money and Mental Health Policy Institute^d^ | UK survey of 568 adults with experience of mental health problems  Conducted between the 20^th^ of March and 30^th^ of March | 86% of the survey respondents were worried about accessing mental health services if needed. Regarding financial issues:   - 57% were worried about losing their job due to the pandemic - 62% were worried about having access to the benefits system - 56% were worried about creditors chasing them for money |
| Costa et al (pre-print)^e^ | US research study of an online survey of 193 adults living with mental health problems  Conducted in the last week of March 2020 | 64% of survey respondents fear their mental health will worsen, and 39% are concerned they will not be able to access mental health services. Only 12% of respondents felt they were coping well with the pandemic and public health measures implemented. |
| Hao et al 2020^f^ | Chinese research study of 76 service users and 109 ‘healthy control’ adults  Conducted in late February 2020 | Compared to the general population, service users were experiencing more severe symptoms of PTSD, anxiety, depression, stress and insomnia and had higher suicidal ideation at the height of the pandemic and lockdown measures in China |
| Hafal^g^ | National Welsh survey of over 300 mental health services users  No details published on the timing of the survey or the methodology used | 74% of survey respondents reported negative effects of the coronavirus outbreak on their mental health. 63% had not been able to contact their GP in the last two weeks, and 14% had difficulties in getting hold of their Community Mental Health team |

*^a^YoungMinds. Coronavirus: Impact on young people with mental health needs. 2020. https://youngminds.org.uk/media/3708/coronavirus-report_march2020.pdf (accessed May 29, 2020)*

*^b^Rethink Mental Illness. 80% of people living with mental illness say current crisis has made their mental health worse. 2020 https://www.rethink.org/news-and-stories/news/2020/04/80-of-people-living-with-mental-illness-say-current-crisis-has-made-their-mental-health-worse/ (accessed May 29, 2020)*

*^c^The Academy of Medical Sciences. Survey results: Understanding people’s concerns about the mental health impacts of the COVID-19 pandemic. 2020. https://acmedsci.ac.uk/file-download/99436893 (accessed May 29, 2020)*

*^d^D'Arcy C. Money and mental health at a time of crisis. 2020 https://www.moneyandmentalhealth.org/wp-content/uploads/2020/04/Money-and-mental-health-coronavirus-policy-note.pdf (accessed May 29, 2020)*

*^e^Costa M, Pavlo A, Reis G, Ponte K, Davidson L. Covid-19 concerns among persons with mental illness (pre-print). Psychiat Serv 2020; 1–5.*

*^f^Hao F, Tan W, Jiang L, et al. Do psychiatric patients experience more psychiatric symptoms during COVID-19 pandemic and lockdown? A case-control study with service and research implications for immunopsychiatry. Brain Behav Immun 2020; S0889-1591(20)30626-7. doi: 10.1016/j.bbi.2020.04.069.*

*^g^Hafal. Survey raises concerns about the provision of mental health services in Wales during the Covid-19 outbreak. 2020 Survey raises concerns about the provision of mental health services in Wales during the Covid-19 outbreak (accessed May 29, 2020)*

**Experiences of people with mental health problems**

## **Table 3:** *Loneliness, social isolation and loss of routines and activities*

**Total unique sources:** 230

**Theme summary:**

Mental health symptoms were significantly affected by increased feelings of isolation and loneliness. Themes emerged around the detrimental effects of disruptions to routines, and loss of contact with friends and family. Many people relied on routines and social contacts to manage their mental health condition, feel connected, and detect signs of deterioration, and the loss of this led to increased loneliness. Closure of community services, and therapeutic activities and groups produced similar effects- many reported heavy reliance on peer and support groups. Patients in inpatient settings were particularly affected by suspension of visitation and leave, leading to extreme isolation and loneliness. This was compounded further for many by instruction to stay confined within rooms and cancellation of activities and forms of social contact. Confinement and increased isolation and loneliness also arose in some cases where living conditions were adverse, physically or psychologically. There were also a few positive impacts of lockdown reported, described as “normalisation” of usually solitary life, and the ability to more easily adapt to global social isolation when accustomed to solitude. However, these effects were potentially transient, and there was concern around longer-term negative impact.

| *Theme* | *Number of sources* | *Countries* | *Author types* | *Source type* | *Content* |
| --- | --- | --- | --- | --- | --- |
| Loneliness and isolation impact on mental health | 175 | UK (32), USA (26), International (25), Italy (25), France (18), Spain (6), Canada (4), Switzerland (4), Australia (2), Belgium (2), China (2), Austria (1), Costa Rica (1), Indonesia (1), Ireland (1), New Zealand (1), Peru (1), | Journalist (100), Clinician (30), Scientist (24), Policy, professionals and charity sector (18), Person with relevant lived experience (15), Unknown (7) | Media article (90), Organisational website (35), Specialist health and social care press (21), Journal article (13), Video/Webinar/Podcast (7), Policy body (5), Blog (4) | Many sources reported the negative impact of loneliness and isolation on mental health in people already living with a mental health condition. These mentioned the impact of lockdown and the different ways this affected people, such as the lack of social contact, the disintegration of usual support structures, and having nothing to distract them from their thoughts, leading to increased feelings of loneliness and isolation. Some mentioned consequences such as an increase in alcohol consumption or online gambling. There were reports on the differential ways that isolation impacted on different mental health conditions, such as depression, anxiety, OCD, and eating disorders. The tensions between years of therapeutic work focused on making more social contacts and the requirements of “lockdown” were discussed by some. |
| Disruption to routines and activities | 81 | UK (15), Italy (14), France (11), Germany (11), International (9), USA (9), Spain (4), China (2), Australia (1), Austria (1), Belgium (1), Indonesia (1), Qatar (1), Switzerland (1) | Journalist (43), Person with relevant lived experience (15), Clinician (13), Policy, professional and charity sector (6), Scientist (5), Unknown (4) | Media article (47), Organisational website (16), Specialist health and social care press (9), Video/Webinar/Podcast (5), Journal article (2), Blog (1), Policy body (1) | The negative effects of disruption to usual routines and activities were frequently discussed. Many people reported finding the days empty and lacking structure, activities, and social contact and that this made them feel even more isolated with a worsening effect on their mental health. There were many first-person accounts of how this disruption was affecting people with specific conditions, such as eating disorders, depression, and OCD. People often reflected on how much they had relied on these routines and activities to manage their mental health, with greater risk of relapse as a result. |
| Impact of lack of seeing family/friends/social contact | 34 | UK (16), USA (5), International (4), Spain (2), Belgium (1), Canada (1), France (1), Germany (1), Ireland (1), Italy (1), New Zealand (1) | Journalist (14), Person with relevant lived experience (11), Policy, professional and charity sector (6), Clinician (3), Unknown (2), Scientist (1) | Media article (18), Organisational website (13), Journal article (2), Video/Webinar/Podcast (1) | Linked to disruption to activities, there was considerable specific mention of the detrimental effects of not being able to see family and friends, and other forms of in-person social contact. As well as making people feel more isolated and lonelier, there were many reports of how much some people relied on those around them to monitor and detect when mental health symptoms were worsening. People reported that telephone or online contact did not necessarily replace these essential support mechanisms and that the nuances of communication were often lost in these alternative communication formats. Some people described how hard they had worked to develop and manage healthy social contact as a way out of isolation and that the impact of lockdown was a huge backward step. |
| Impact of closed mental health facilities, therapeutic activities and support groups | 41 | Italy (11), France (8), UK (7), USA (5), Germany (3), International (3), Spain (2), Belgium (1), Switzerland (1) | Journalist (19), Policy, professional and charity sector (9), Person with relevant lived experience (6), Clinician (5), Scientist (3), Unknown (1) | Media article (21), Organisational website (10), Specialist (4), Journal article (3), Policy body (2), Video/Webinar/Podcast (1) | There were a number of reports about the negative effects of closure of mental health services, therapeutic activities and support groups in the community, and the resulting increased sense of isolation and loneliness. This was, for example, a prominent theme in Italian reports, where many service users were frequently attending community mental health centres where employment, activity, social contact and treatment were offered. The effects varied from having a lack of distraction and routine, feeling cut-off from usual support sources and structures, and an exacerbation of ruminations and worries. Many people reported how much they relied on peer or other types of support groups, such for recovering alcoholics, and people with OCD. |
| Social impact of lack of visits and lack of social contact in inpatient/residential settings | 28 | Italy (7), France (4), International (4), UK (4), USA (4), Switzerland (2), Costa Rica (1), Germany (1), Spain (1 | Journalist (18), Scientist (4), Clinician (3), Policy, professional and charity sector (2), Person with relevant lived experience (1) | Media article (17), Organisational website (4), Journal article (3), Specialist health and social care press (2), Blog (1), Video/Webinar/Podcast (1) | Some sources explored the isolation of people in inpatient and some residential settings following the suspension of visits from friends and family, and on external leave by residents. There were many reports of people feeling isolated as a result of the loss of contact with loved ones and an exacerbation of mental health conditions, including suicidal ideation and self-harm. There was specific mention of the significant impact on children and young people, and on people with a schizophrenia diagnosis. One source described residents who could not understand why they were no longer receiving visitors.  There were also reports of people not having access to digital means of contact from hospital or not being allowed to use the telephone on infection grounds. Reduced contact with staff and other patients was described as further adding to loneliness and boredom in inpatient settings. |
| Confinement in inpatient facilities and lack of activities | 13 | France (4), UK (3), USA (3), Italy (2), International (1) | Journalist (7), Clinician (3), Scientist (2), Policy, professional and charity sector (1) | Media article (9), Journal article (1), Organisational website (1), Specialist health and social care press (1), Video/Webinar/Podcast (1) | There were also several accounts of the impact of a combination of confinement and suspension of usual activities in inpatient or residential settings, with a loss of therapeutic aspects of wards stays, and challenging experiences of boredom and isolation when confined to rooms all day. |
| Adverse living conditions (small, chaotic, family tension, domestic violence) | 8 | France (2), Italy (2), Indonesia (1), International (1), New Zealand (1), UK (1) | Journalist (3), Clinician (2), Unknown (2), Policy, professional and charity sector (1) | Media article (3), Organisational website (2), Journal article (1), Policy body (1), Video/Webinar/Podcast (1) | A few reports described the exacerbation of feelings of isolation and loneliness in people as a result of challenging living conditions, such as living in cramped or chaotic spaces, or where family tensions were already high. |

## **Table 4:***Access to essential services & resources: reports on individual experiences*

**Total unique sources: 225**

**Theme summary:**

A series of challenges were identified relating to individual experiences of access to services and resources. Many sources described a reduction in available health and social care due to COVID-19 and corresponding restrictions. This was apparent for various types of mental and physical health problems and formal and informal care types, with fears about resulting mental health deterioration and future strain on services. There were concerns about COVID-19 magnifying existing inequalities and creating new ones, with the impact of COVID-19 and restrictions seen as disproportionality affecting those already experiencing social inequalities, exacerbating existing social inequalities, and creating new inequalities and underserved groups. There was particular concern that individuals from underserved groups would have greatest problems with access to food and essential goods, support, resources and money, physical and mental healthcare, and COVID-19 testing and treatment, and that individuals with pre-existing mental health problems would be disproportionally affected by unemployment. There were also concerns about infection and overstretched services reducing help-seeking, which interplayed with mental health stigma, potentially contributing dual barriers to help-seeking. Historic underfunding and a lack of services in some areas, resulting in high levels of unmet need (especially for early intervention) and upstream strain on healthcare services (e.g., crisis care), was also seen as potentially being magnified due to COVID-19. Disruption to medication access and adherence was also seen as affecting management of mental health problems, while self-medication, addictions, and worsening mental health were seen as increasing health risk behaviours (especially alcohol and tobacco) in response to the strain of the pandemic.

| *Sub-theme* | *Number of sources* | *Countries* | *Author types* | *Source types* | *Content* |
| --- | --- | --- | --- | --- | --- |
| Lack of access to mental health and other types of service | 120 | UK (36), USA (18), France (14), Italy (13), China (11), International (11), Spain (5), Germany (3), Algeria (1), Argentina (1), Australia (1), Canada (1), Indonesia (1), Mexico (1), New Zealand (1), Singapore (1), Switzerland (1) | Journalist (49), Clinician (24), Scientist (22), Policy, professional and charity sector (18), Person with relevant lived experience (15), Unknown (4) | Media article (55), Organisational website (29), Journal article (16), Specialist health and social care press (10), Blog (4), Video/Webinar/Podcast (3) | A common theme was the reduction in available health and social care due to COVID-19. In some areas only urgent response care remained open (for example in some areas of Italy and Spain in the early period of the pandemic). Abrupt terminations of planned programmes of therapy, withdrawal of help for newly identified problems, and very late intervention in mental health crises because of lack of other responses were also described. When services were running, several sources reported that they were running at reduced capacity or limited times, or service users were uncertain which services were available, or how help might be sought. Sources also reported that people with mental health problems were disproportionately affected by reduced services in primary and social care, and the withdrawal of a wider range of services such as libraries and community centres. |
| "We are not all in this together": COVID-19 magnifying existing inequalities and creating new ones | 79 | UK (19), USA (14), France (11), Italy (11), Germany (8), International (8), China (3), Australia (1), Austria (1), Ghana (1), New Zealand (1), Spain (1) | Journalist (30), Clinician (25), Scientist (15), Policy, professional and charity sector (12), Person with relevant lived experience (10), Unknown (2) | Media article (33), Organisational website (21), Specialist health and social care press (10), Journal article (7), Policy body (4), Blog (2), Video/Webinar/Podcast (2) | A common theme was that "We are not all in this together": COVID-19 ls magnifying existing inequalities and creating new ones. COVID-19 and accompanying restrictions were seen as disproportionality affecting those already experiencing social inequalities including people with long-term mental health problems. The predominant groups at risk of magnified inequalities discussed were the economically disadvantaged, those with residential uncertainty, minority ethnic, and multiple at-risk groups (e.g., homeless individuals with mental health problems or substance use already experiencing challenges accessing food and accommodation). Multiple sources expressed a concern that individuals from already underserved groups, such as people with long-term mental health conditions, would have least access to food and essential goods, support, resources and money, physical and mental healthcare, and COVID-19 testing and treatment, magnifying inequalities already present. |
| Insufficiency of remote services | 34 | UK (9), USA (8), International (5), Germany (3), France (2), Italy (2), Argentina (1), Australia (1), China (1), Indonesia (1), Spain (1) | Journalist (20), Clinician (7), Scientist (7),Policy, professional and charity sector (4), Person with relevant lived experience (3) | Media article (18), Specialist health and social care press (5), Journal article (4), Organisational website (4), Blog (1), Policy body (1), Video/Webinar/Podcast (1) | A number of sources described their difficulties in getting access to health and other services provided by remote or digital means. Reasons given included lack of technology access for certain groups (e.g., economic disadvantage, residential uncertainty, older age, neurocognitive impairment), some insurance providers not paying for remote services or it being available only via private providers, unreliable connections, short consultations due to high demand, lack of privacy in the home for sensitive conversations, certain care being impractical remotely (e.g., physiotherapy, group sessions), and continuity of care when transitioning form face-to-face services.  On the other hand, there were also reports regarding people who are technologically well-connected but struggle to go out in the community and/or experience social anxiety who have found services and the social world more accessible and benefited from access to a wide range of online resources and services. |
| Medication access | 22 | UK (7), China (6), International (2), USA (2), France (1), Germany (1), India (1), Italy (1), Spain (1) | Journalist (7), Policy, professional and charity sector (6), Person with relevant lived experience (4), Clinician (3), Scientist (3) | Media article (7), Organisational website (5), Blog (3), Journal article (3), Policy body (2), Specialist health and social care press (1), Video/Webinar/Podcast (1) | Disruptions to medication access resulted from lack of face-to-face appointments for prescription writing, administration of depots and monitoring, as with lithium and clozapine, loss of contact with services, and also delays in pharmacies and medication ordering, and unexpected relocations, as from university to home. Where a very strict lockdown was in place, as in China, some reported that they and their families found it very difficult to travel to obtain medication. |
| Hesitation to seek help | 17 | UK (9), Germany (3), Indonesia (1), International (1), Italy (1), New Zealand (1), USA (1) | Journalist (8), Policy professional and charity sector (4), Clinician (2), Person with relevant lived experience (1), Scientist (1), Unknown (1) | Media article (8), Organisational website (5), Specialist health and social care press (3), Blog (1) | This theme pertained to concerns about infection and overstretched services reducing help-seeking even where people were aware that services remained open. For example, a survey of individuals with mental health problems found that 36% of respondents had not booked or attended medical appointments because appointments were not available or because of concern about infection and overstretched services. Some also expressed concerns that over-stretched physical health services, such as in A&E departments, might be less receptive than usual to people with mental health conditions, for example seeking help following self-harm, or that in a crisis the physical health needs of people with mental health problems would be taken less seriously. |
| Pressures on already stretched services | 16 | UK (4), USA (4), Italy (3), Australia (1), France (1), Germany (1), International (1), Spain (1) | Clinician (5), Journalist (5), Scientist (3), Policy, professional and charity sector (2), Person with relevant lived experience (1), Unknown (1) | Media article (6), Organisational website (5), Journal article (2), Specialist health and social care press (2), Blog (1) | Some sources described the pandemic as contributing to the cycle of unmet need and strain on healthcare services for mental health, referring to historic under-funding and inequitable provision of mental healthcare. The priority currently given to physical healthcare during the pandemic, even to the extent of closing some mental health services in some areas, was seen as understandable, but also magnifying a long-standing inequity between physical and mental health care. |
| Increased experiences of economic hardship and lack of basic resources | 8 | UK (4), USA (3), Italy (1) | Journalist (4), Policy, professional and charity sector (2), Scientist (1), Unknown (1) | Media article (4), Organisational website (3), Specialist health and social care press (1) | Other sources described the pandemic compounding economic hardship and poverty, especially through loss of employment and where prices for basic resources for food had risen. |

## **Table 5:***Family and social adversities and safety concerns*

**Total unique sources: 110**

**Theme summary:**

The pandemic is a challenging period for many family members and carers of people with mental health problems. The reduction in support from mental health services, combined with reduced face to face contact with family and friends, means that many people are concerned about the mental wellbeing of vulnerable relatives. Reductions in provision of both community and inpatient services means that families have more responsibility for caring for service users. In many cases, they feel like they have been left to do this by themselves. However, families and carers are also experiencing less support from groups and other services. This combined with being in lockdown means that some service users felt greater anxiety or depression due to worries about being a burden on their relatives. A persistent concern, increasing anxiety across all groups, was that relatives may become infected with the coronavirus, particularly those at greater risk of severe illness. More remote and digital support has been developed, with some service users and carers reporting developing new support networks online or keeping in contact with friends and family by phone or online. This was seen as a positive development by many service users especially, and something they hoped would continue once the lockdown ends.

The lockdown has also meant that there is widespread concern about the likely increase in conflict, aggression, and potentially violence between household members and especially towards children. These difficulties may be exacerbated by increased use of alcohol, online gambling, or substances at home, and in the USA, there are reports of a substantial increase in gun sales. Furthermore, people living in poverty and poor social circumstances, including many people with severe mental health illnesses, are likely to be at higher risk of adverse events. Several authors were concerned that limited availability of the courts, police and social services may impede abusive situations being addressed: novel ways of communicating with people at risk, such as via social media, have been discussed.

| *Sub-theme* | *Number of sources* | *Countries* | *Author types* | *Source types* | *Content* |
| --- | --- | --- | --- | --- | --- |
| Strain on family and carers | 55 | Italy (19), France (9), UK (9), USA (5), Germany (3), Spain (3), International (2), Costa Rica (1), India (1), Ireland (1), New Zealand (1), Singapore (1) | Journalist (29), Clinician (13), Policy, professional and charity sector (8), Person with relevant lived experience (6), Scientist (4), Unknown (1) | Media article (29), Organisational website (11), Specialist health and social care press (5), Journal article (4), Policy body (3), Video/Webinar/Podcast (2), Blog (1) | The current lack of support from mental health services mean that family members, and especially members of the same household, take on more of a role as carer and may have to handle complex mental health issues on their own. In some countries, such as France and Germany, beds were being freed up in psychiatric hospitals for coronavirus patients by hospitals refusing to admit psychiatric patients except for in extreme cases and by discharging more stable inpatients into the care of their families. Meanwhile, families/carers have to make do with less support from mental health services (family groups, carer support, and other forms of support). Some families reported feeling ‘abandoned’.  There were also reports from carers and family members that they were worried about vulnerable relatives because of reduced care from mental health services, isolation, other impacts on mental health.  Finally, there were concerns regarding children of mental health service users, as families are more isolated and less supported than usual and safeguarding mechanisms may not be operating as usual. |
| Increased risk of violence | 36 | Germany (11), France (6), UK (6), USA (5), Italy (4), International (3), Spain (1) | Journalist (18), Policy, professional and charity sector (9), Clinician (6), Scientist (6), Unknown (1) | Media article (19), Specialist health and social care press (6), Organisational website (5), Journal article (3), Policy body (2), Video/Webinar/Podcast (1) | A widespread concern is that confinement risks increasing conflict, aggression, and potentially violence between household members and especially towards children. Reasons for this identified risk in the families of people living with mental health conditions include interpersonal tensions from spending a lot of time together, some symptoms of mental illness, evidence that alcohol use and gambling may have increased for some, stress from fear of unemployment, feeling overwhelmed with juggling work and teaching children at home, school closures, and not having the usual support from friends or family. People with mental health conditions may be at risk both as victims and as perpetrators. |
| Concerns about family relationships | 22 | UK (5), France (4), International (3), USA (3), Germany (2), Italy (2), China (1), Spain (1), Switzerland (1) | Journalist (9), Clinician (5), Person with relevant lived experience (4), Policy, professional and charity sector (4), Scientist (4), Unknown (1) | Media article (10), Organisational website (7), Journal article (3), Blog (1), Specialist health and social care press (1) | There were reports from service users that strained relationships while confined with family members were having negative effects on their mental health. Others reported worrying about being a burden on family members with whom they were staying, or about transmitting COVID-19 to them, especially if they were physically vulnerable. Loss of contact with family members, as above in the section on Loneliness and Social Isolation, was an important concern for others. Some service users, and especially people with eating disorders, found it difficult to be isolated with family members because it was more difficult to hide their illness from relatives. |
| Support for families | 10 | UK (3), France (2), USA (2), Belgium (1), International (1), Italy (1) | Policy, professional and charity sector (4), Journalist (3), Clinician (2), Person with relevant lived experience (2), Journalist (2), Scientist (1) | Media article (4), Organisational website (3), Journal article (1) Policy body (1), Specialist health and social care press (1) | Lockdown measures impact on families, carers, and service users in various ways, including increasing the risk of relapse or significant safeguarding challenges, and authors identify a need to develop new strategies for helping at risk populations: for example people experiencing domestic violence and children at risk may find it difficult to seek help when family members are always there, so that channels such as social media may be valuable.  Meanwhile, the lack of support from services for families’ worries about vulnerable relatives has led to some service users moving in with family to reduce isolation and improve care and obtain more support, or were communicating more with family and friends by remote or online methods, or developing new support networks by this means. |

## **Table 6:** *COVID infection risks and risks of severe illness for people living with mental health problems*

**Total unique sources: 158**

**Theme summary:**

At this stage in the pandemic, we did not find data on the prevalence or severity of COVID-19 infection or many accounts of experiences of having the infection among people living with mental health problems. There were concerning accounts of outbreaks in psychiatric or residential settings in several countries, with discussions of infection control problems that may make this more likely: see below for further discussion of outbreaks in inpatient settings.

Sources reported that some people with mental health problems are at increased risk of severe COVID-19 infections because of physical comorbidities, unhealthy lifestyles (e.g., obesity and smoking), and potentially medication effects. Due to confined, crowded, or chaotic living environments individuals with mental health problems (especially those who are homeless) may also be at greater risk of infection of COVID-19.  According to some sources, some individuals with mental health problems may also experience difficulties with practicing infection control measures, due to difficulties in understanding the measures and why they are needed, low risk awareness, and reduced attention to protection measures. One theme describes mental health stigma as a barrier to accessing treatment for COVID-19, interfering with the ability of service users to receive equitable health care and, in the extreme, worse health outcomes and mortality when experiencing COVID-19. A significant number of sources focused on health anxiety related the virus, as already described in Table 2.

| *Sub-theme* | *Number of sources* | *Countries* | *Author types* | *Source types* | *Content* |
| --- | --- | --- | --- | --- | --- |
| Challenges for people with mental health problems in following infection control measures | 71 | France (17), Italy (13), Germany (9), UK (9), International (8), China (4), USA (4), Spain (3), Switzerland (2), Belgium (1), Ghana (1) | Clinician (29), Journalist (27), Scientist (15), Policy, professional and charity sector (7), Unknown (6), Person with relevant lived experience (4) | Media article (25), Specialist health and social care press (13), Journal article (11), Organisational website (11), Video/Webinar/Podcast (6), Policy body (5) | A common theme was cognitive, behavioural, environmental, or self-protection impairment making infection control challenging in people with mental health conditions. Importantly, understanding and applying hygiene and social distancing rules and maintaining sustained attention to these was described as particularly challenging for people who are cognitively impaired, distressed or symptomatic, or who are using substances heavily. Living in confined, crowded, or chaotic settings (e.g., incarceration, inpatient or residential, homeless or residential uncertainty) was described as posing an important environmental impairment making infection control challenging and potentially increasing the risk of infection; some sources noted this was exacerbated by inadequate infection control equipment in organisations and the potential of staff to bring the virus in to closed institutions from the outside, with a smaller number of pieces commenting on the risk of staff contracting the virus from patients.  Lack of guidance that is tailored to people with cognitive impairments, or to other barriers to understanding, was an important theme in some discussions. Some pieces noted that a lack of understanding of these impairments by authorities has meant that non-adherence to infection control in some instances has escalated to the point of police involvement. Moreover, some pieces noted the need to adhere to infection control in a manner that minimizes psychological distress; for example, individuals with cognitive impairment who do not understand why staff are wearing personal protection equipment. |
| Experience of outbreaks | 10 | Italy (3), USA (2), China (2), Columbia (1), International (1), Spain (1) | Journalist (6), Person with relevant lived experience (2), Clinician (1), Scientist (1) | Media article (5), Specialist health and social care press (4), Blog (1) | This theme describes the experience of outbreaks of COVID-19 in service users and staff in residential, inpatient, and psychiatric hospital settings, including some concerning accounts of difficulty in containing spread within psychiatric inpatient units. There was mention of inadequate infection control measures in these settings as contributing to these outbreaks, especially when mental health patients could not be transferred to general hospitals. The challenge of maintaining adequate mental and physical healthcare during outbreaks in these settings was also briefly discussed. |
| Increased health anxiety related to COVID-19 | 48 | UK (14), USA (10), Germany (5), Spain (4), France (3), Italy (2), Argentina (1), Australia (1), Canada (1), China (1), Colombia (1), Dominican Republic (1), International (1), Mexico (1), Peru (1), Switzerland (1) | Journalist (25), Person with relevant live experience (13), Clinician (8), Scientist (6), Policy, professional and charity sector (1), Unknown (1) | Media article (30), Organisational website (9), Journal article (4), Specialist health and social care press (2), Video/Webinar/Podcast (2), Blog (1) | A large proportion of sources reported increased health anxiety among people with pre-existing mental health problems, as already discussed above in Table 2, with many reporting anxieties about contracting or spreading the virus, or about family members becoming unwell. Individuals, particularly with pre-existing mental health problems have described high levels of concern about contracting the virus, family members contracting the virus, or spreading the virus, and interactions especially with OCD and health anxiety related to contamination. However, other sources describe coping relatively well with concerns around the pandemic despite existing health anxieties or OCD in which fears regarding health have a different focus. Other reported fears related to uncertainty about how long the pandemic and its associated risks and restrictions. |
| Increased risk of being infected, or of infection being severe | 44 | France (10), UK (9), International (8), USA (7), China (5), Australia (1), Colombia (1), India (1), Italy (1), Spain (1) | Clinician (16), Scientist (15), Journalist (12), Person with relevant loved experience (4), Policy, professional and charity sector (4), Unknown (4) | Media article (13), Journal article (10), Organisational website (8), Specialist health and social care press (6), Policy body (4), Blog (2), Video/Webinar/Podcast (1) | A large proportion of sources discussed potentially increased risks of infection, or of severe infection from COVID-19 for people with mental health problems, although without data so far being available regarding rates of COVID-19 infection and of severe illness associated with this among people with severe mental illness, or of experiences of this illness.  Factors potentially resulting in more severe illness from COVID-19 included comorbid physical health conditions, lifestyle problems (e.g., obesity, smoking) or poorer overall physical health conferring greater risk of severe illness. Individuals with eating disorders suffering from malnutrition and compromised immune systems may be at greater risk of severe illness, and other sources discuss risks associated with substance misuse, such as on the lung function and the brain. Factors potentially increasing risk of being infected and/or making illness harder to manage include homelessness, over-crowded or chaotic living circumstances, and incarceration, all more frequent among people with mental health problems. |
| Interactions between COVID-19 and multiple needs, disadvantages and competing mental and physical health problems | 44 | France (13), International (7), UK (6), USA (5), China (4), Spain (4), Italy (2), Colombia (1), Germany (1) | Clinician (18), Journalist (17), Scientist (17), Person with relevant lived experience (4), Policy, professional and charity sector (3), Unknown (3) | Media article (15), Journal article (10), Organisational website (8), Specialist health and social care press (7), Blog (3), Policy body (1) | This theme pertains to the competing mental and physical health needs of experiencing mental health problems and experiencing (either directly or in the household) COVID-19. Fpr example, one source discussed the challenges of having the virus whilst being alone. Another described mental health remaining the biggest concern of an individual despite having the virus. One case presentation described an individual who found it difficult to discriminate between COVID-19 symptoms and panic attack symptoms. Two pieces described the challenges of experiencing exacerbated mental health difficulties while caring for someone with COVID-19 in the household and corresponding challenges, such as being ordered back to work.    Some also noted that individuals with mental health problems may have greater difficulty expressing and seeking help for their physical health needs. Similarly, health inequalities mean individuals from economically disadvantaged backgrounds may have higher risk of mental and physical health problems in general and infection or poor outcomes related to COVID-19 in particular. |
| Stigma as a barrier to treatment | 12 | Italy (4), France (3), USA (2), Argentina (1), Germany (1), UK (1) | Clinician (5), Journalist (5), Scientist (3), Person with relevant lived experience (1), Policy, professional and charity sector (1) | Journal article (3), Media article (3), Specialist health and social care press (2), Blog (1), Organisational website (1), Policy body (1), Video/Webinar/Podcast (1) | A common theme was concern that individuals with mental health problems might experience “dual stigma” in terms of additional barriers to accessing physical health care for COVID-19 due to stigma and discrimination from healthcare staff who are not specialised in mental health; this was discussed as both a potential concern and an actual issue. Concerns included that people without mental health problems may be prioritised for physical care over those who do not have mental health problems, that COVID-19 care delivered in psychiatric settings may be of lesser quality (discussed further below) and that people with mental health problems treated in general hospitals may not have their mental health needs sufficiently addressed. However, some sources did describe guidance put in place with the aim of ensuring equitable treatment for people with mental health problems becoming ill from COVID-19 infection. |

## **Table 7:** *Positive experiences of life in the pandemic period*

**Total unique sources: 113**

**Theme summary:**

 A number of positive aspects of life during the pandemic were reported. Some drew comfort from the fact that everyone was ‘in the same boat’, with the rest of society now experiencing similar problems to their own. Feelings of decreased marginalisation, greater acceptance, and increased levels of community and solidarity were reported. There were accounts of hitherto unrecognised reserves of resilience and coping skills. Some have taken the opportunity to pursue other worthwhile activities, and to reflect on their priorities and life goals. The pandemic has enabled some to increase or decrease their desired levels of contact with others. Improvements in symptom severity and in experiences of receiving mental health services, particularly the adoption of online consultations, were also reported.

| *Sub-theme* | *Number of sources* | *Countries* | *Author types* | *Source types* | *Content* |
| --- | --- | --- | --- | --- | --- |
| Coping well with lockdown measures | 64 | USA (16), Germany (11), UK (11), Italy (9), France (7), International (5), Spain (3), Australia (1), Switzerland (1) | Journalist (41), Person with relevant lived experience (16), Clinician (11), Policy, professional and charity sector (2), Unknown (2), Scientist (1) | Media article (43), Organisational website (11), Specialist health and social care press (8), Policy body (1), Video/Webinar/Podcast (1) | There were many reports of service users feeling relatively well prepared to deal with the current situation because it suits their normal habits/lifestyle. Some clinicians expressed surprise at how well some of their patients had adapted to the pandemic.  Enforced isolation can be “a blessing in disguise” because it allows people to avoid social contact. They don't have to worry about certain errands or tasks that they normally find difficult, such as going to banks. One OCD patient said that they feel better knowing everyone is washing their hands and that their anxieties have been validated.  They felt that their coping skills, acquired over many years, enabled them to handle the pandemic well. Others felt that the situation had encouraged them to develop new, effective coping strategies that might prove beneficial in the future.  Meanwhile, the situation had created a ‘level playing field’ because everyone was staying at home. Some service users said their relationships and levels of contact with friends and family were now better than before because of increased use of videocalls. |
| Shared experiences and solidarity | 19 | Italy (8), Germany (3), UK (3), International (2), Australia (1), Austria (1), USA (1) | Journalist (11), Clinician (4), Person with relevant lived experience (2), Scientist (2), Policy, professional and charity sector (1), Unknown (1) | Media article (10), Organisational website (4), Specialist health and social care press (3), Blog (1), Journal article (1) | Some viewed the pandemic as a ‘shared trauma’ that had created a greater awareness and acceptance of mental health issues in the general population. This was especially true in accounts from people with OCD/anxiety. Some gained comfort from the fact that certain behaviours had now become normalised.  Feelings of greater acceptance, belonging, and increased understanding and empathy among the wider community were welcome. Some reports described this shared experience as creating a growing sense of community or solidarity between those with mental health problems and the general population.  However, doubts were expressed about whether this increased understanding would persist beyond the duration of the pandemic. |
| Improvement in mental health | 13 | UK (3), France (2), International (2), Austria (1), Canada (1), Germany (1), Italy (1), Switzerland (1), USA (1) | Journalist (8), Clinician (2), Person with relevant lived experience (2), Policy, professional and charity sector (1), Unknown (1) | Media article (8), Specialist health and social care press (3), Organisational website (2) | Reduction in symptoms of pre-exiting conditions (including depression, OCD, social anxiety, mood disorders, and bulimia) was reported in a number of sources.  In many cases, this was because they felt less pressure, for example to socialise or to run errands. There were many reports of a significant reduction in work, school, and other stresses, and improved sleep as a result. But some also described an improvement to mental health because people were distracted by the pandemic.  For some, coping well had shown them how far they had come or how resilient they were. Covid-19 is not as worrying for some patients as other issues. However, feelings of guilt for having these positive experiences were also reported. |
| Improvements to support | 20 | Italy (7), UK (4), International (3), USA (3), Germany (2), France (1) | Journalist (10), Clinician (6), Person with relevant lived experience (3), Scientist (2), Policy, professional and charity sector (1) | Media article (6), Specialist health and social care press (6), Organisational website (4), Video/Webinar/Podcast (3), Journal article (1 | Some service users welcomed the move to online consultations resulting from the pandemic, preferring them to their face-to-face equivalent.    Meanwhile, some of the other initiatives that have been set up to help vulnerable people, such as home shopping deliveries, are also very welcome, with some people feeling better supported in their communities than before. |
| More time | 7 | Germany (2), UK (2), USA (2), France (1) | Journalist (4), Person with relevant lived experience (3), Clinician (1), Scientist (1) | Media article (3), Organisational website (3), Video/Webinar/Podcast (1) | The pandemic has provided some with an opportunity to spend more time with family, which can be an important source of support.  One source reported more time for new leisure activities, such as walking in nature, which they find therapeutic. A clinician working within an inpatient setting reported patients doing more activities and games.  Some with lived experience welcomed the opportunity provided by isolation to pause, reflect and evaluate their priorities and life goals. Others reported helping others and contributing to the wider community (e.g. making face masks, volunteering). |

**Individual coping strategies in the times of the pandemic**

## **Table 8a:***Strategies people with lived experience employ to manage life in pandemic period*

**Total unique sources: 97**

**Theme summary:**

Many people reported taking positive steps to improve or maintain their health and wellbeing, especially since the usual opportunities for doing so were now limited. In this section, we report strategies that people with relevant personal experience themselves describe making use of (themselves or via interviews with journalists): these are often in the context also of making suggestions to others: clearly we cannot say how representative these authors are in their approach to managing their mental health problems. Strategies include engaging in purposeful, creative or relaxing activities, such as cooking, painting, watching television and listening to music. Some reported keeping a journal to record their worries or positive experiences. Use of therapeutic and self-help techniques, such as mindfulness, exposure therapy, yoga and meditation, was widely reported, though some found these of limited use. The importance of maintaining a positive attitude, of self-acceptance and of not putting oneself under pressure was widely expressed. Looking after one’s physical health, such as taking regular exercise and healthy eating, and maintaining contact with trusted friends and family members, was emphasised in many sources. Maintaining a daily routine to reduce rumination and boredom was frequently recommended. A number of people, particularly those with anxiety, reported attempting to avoid or substantially reduce their consumption of potentially stressful or triggering media coverage of the pandemic, relying instead on official or other trusted sources. A range of self-management tools and resources, including helplines, online therapy services, websites, podcasts and apps, were found to be helpful. Few sources reported on coping strategies used by carers or family members.

| *Sub-theme* | *Number of sources* | *Countries* | *Author types* | *Source types* | Content |
| --- | --- | --- | --- | --- | --- |
| Maintaining health and wellbeing | 68 | UK (31), USA (15), Germany (4), France (3), International (3), Italy (3), Switzerland (3), Canada (2), Mexico (2), Australia (1), Spain (1) | Person with relevant lived experience (40), Journalist (30), Clinician (5), Scientist (2), Policy, professional and charity (1), Unknown (1) | Media article (39), Organisational website (16), Video/Webinar/Podcast (7), Specialist health and social care press (5), Journal article (1) | Many people reported taking positive steps to improve or maintain their health and wellbeing, especially since the usual opportunities for doing so were often unavailable during self-isolation. Many activities were undertaken to reduce anxiety.  Purposeful activities included cooking, gardening, home improvements and learning something new. Some reported undertaking creative activities such as writing, painting and composing songs. Distraction or relaxation activities included reading, watching television, listening to music and playing games.  Some reported keeping a journal to record their worries and fears, intrusive thoughts or the positive things that had happened during the day.  Use of therapeutic and self-help techniques (described by some as their “therapy toolkit”), often acquired during previous therapy, was widely reported. This included mindfulness, breathing exercises, exposure therapy, acceptance and commitment therapy, body scanning, yoga, meditation and prayer. Some, however, reported that their self-help activities were of limited usefulness under current circumstances. A small number of sources stressed the importance of continuing to take prescribed medication and of maintaining regular contact with mental health services.  The importance of maintaining a positive psychological attitude was widely expressed. Reference was made to staying optimistic, focusing on positive aspects of one’s situation (such as more time or reduced commitments or interaction with others), having a sense of gratitude for one’s health, family or financial security, gaining a sense of perspective (e.g. regarding the risk of becoming infected), and recognising that many others were in a similar position.  Many stressed the importance of self-acceptance, being kind to oneself, avoiding self-judgment and not putting oneself under pressure to achieve unrealistic “lockdown goals”.  The importance of maintaining physical health, such as regular outdoor physical exercise, adequate sleep, healthy eating and regular meals, and of controlling alcohol and caffeine intake, was stressed by many. Advice on the importance of infection control and personal hygiene was provided in a small number of sources. |
| Social contact | 34 | UK (18), Germany (5), USA (5), Switzerland (3), Canada (1), International (1), Italy (1) | Person with relevant lived experience (21), Journalist (15), Clinician (2), Policy, professional and charity sector (2) | Media article (20), Organisational website (7), Specialist health and social care press (5), Video/Webinar/Podcast (2) | Given the potential adverse effects of social isolation, maintaining regular contact with friends and family via phone or video calls, SMS or social media services such as Facebook or Instagram was reported as important by many.  Some stressed the importance of identifying trusted members of their family or wider social network with whom they could talk honestly and who could be relied on to listen to their problems and concerns and provide support if needed. Some stressed the value of being in contact with people who were aware of their history. Peer support and its benefits are discussed below.  The survey of young people by Young Minds found that maintaining social contact was beneficial but that respondents felt social media use should be controlled as it can have adverse effects on mental health. |
| Structure & routine | 28 | UK (15), USA (5), Germany (4), International (1), Mexico (1), Spain (1), Switzerland (1 | Person with relevant lived experience (16), Journalist (12), Clinician (3), Policy, professional and charity sector (1), Unknown (1) | Media article (21), Organisational website (4), Video/Webinar/Podcast (2), Specialist health and social care press (1) | The importance of creating a structure for day-to-day life and of maintaining a routine was frequently emphasised, replacing disrupted routines that were previously important to self-management. Stated reasons for this included reducing rumination and boredom, and maintaining physical and mental health during isolation, e.g. by keeping unhealthy eating habits under control, or reducing the frequency of handwashing or cleaning.  Routines were often written down in the form of daily or weekly schedules, eating plans or lists of tasks or goals to be achieved. They included activities relating to working, shopping, eating, getting up, self-care, exercise, watching television and speaking to friends and family. |
| Limiting information | 26 | UK (14), USA (5), France (2), International (2), Germany (1), Spain (1), Switzerland (1) | Journalist (12), Person with relevant lived experience (12), Policy, professional and charity sector (2), Clinician (1), Scientist (1) | Media article (15), Organisational website (7), Video/Webinar/Podcast (3), Journal article (1) | A number of people, particularly those with anxiety, attempted to avoid or substantially reduce their consumption of potentially stressful or triggering media coverage of the pandemic. The media was seen as fixating on numbers of deaths, and on risks that were often exaggerated or sensationalised, rather than providing reassurance. Some individuals were regulating their use of social media and taking steps such as turning off ‘push’ notifications.  Instead, people suggested it is preferable to rely on official or scientific sources or people they trusted to provide them with accurate information, or changes to guidelines.  A UK survey of young people (Young Mind, Table 1) found that 66% of respondents highlighted that watching the news was not helpful and caused further anxiety. |
| Self-management tools | 20 | UK (8), USA (5), Italy (2), China (1), France (1), Germany (1), International (1), Qatar (1) | Journalist (14), Person with relevant lived experience (8), Policy, professional and charity sector (1) | Media article (12), Organisational website (6), Specialist health and social care press (2) | 20 sources reported beneficial use of a variety of self-management tools and resources in order to help them cope with the consequences of isolation, anxiety and other issues.  These included helplines and telephone-based listening services run by e.g. mental health charities; SMS-based support services (e.g. Crisis Text Line); online therapy, diagnosis and treatment services; websites (e.g. https://www.dbtselfhelp.com); podcasts (e.g. “Sleep With Me”); psychoeducation tools; and apps for meditation and mindfulness (e.g. Calm and Headspace).  Some reported online peer support communities (e.g. “Clic”) to have been helpful.  Use of mainstream social media apps (e.g. Instagram, Facebook pages) and group-watching services (e.g. Netflix Party) was also reported. One person with lived experience curated a social media feed of accounts geared toward recovery. |
| Carer strategies | 2 | France (1), USA (1) | Clinician (1), Journalist (1), Person with relevant lived experience (1) | Specialist health and social care press (1), Video/Webinar/Podcast (1) | While many sources stressed the importance of carers and family members staying in touch with and providing support to people with mental health problems during the pandemic, very few reported on coping strategies used by carers or family members themselves.  One parent described how they had lowered their expectations of a child with bipolar disorder and showed greater compassion for themselves. Use of online support groups (e.g. supportgroupscentral.com) was recommended.  One source reported on a French study assessing the consequences of isolation on families/carers.) |

## **Table 8b:** *Peer support*

**Total unique sources: 49**

**Theme summary:**

The facilitation and impact of peer support and mutual aid was reported, including informal support from family and friends. The majority of articles reported on types and impact of practical and emotional support, with online and digital technologies underpinning vital support structures, and peers, friends and family offering practical support such as collecting medication. Sharing experiences and stories of mental health management, coping strategies and positive adaptations featured. Digital and online approaches to delivering support had been rapidly, proactively and creatively deployed to facilitate one to one, group and community connections and activities (including recreation and socialising). Communicating and connecting were considered to be vital for reducing social isolation in lockdown, managing mental health, and maintaining relationships with friends, family and peer support networks. The importance of connecting with others in inpatient settings during the pandemic was mentioned. Mutual aid, peer support and befriending appeared to have positive benefits for those offering support, with some reporting that helping and contributing was positive for their mental health and wellbeing. Very few sources referred to support provided by neighbours or the wider local community.

| *Sub-theme* | *Number of sources* | *Countries* | *Author types* | *Source types* | *Content* |
| --- | --- | --- | --- | --- | --- |
| Practical and emotional support | 15 | UK (6), Italy (3), China (2), International (2), USA (2) | Journalist (6), Person with relevant lived experience (5), Clinician (2), Policy, professional and charity sector (2), Scientist (1) | Media article (7), Organisational website (4), Blog (2), Specialist health and social care press (2) | A significant number of sources reported that mutual aid and peer support initiatives offered both practical and emotional support. Service user community and group support facilitated by online and digital means was reported as providing emotional and psychological support for managing the impact of COVID-19 related distress, relationship issues, feelings of loneliness and isolation, symptom management (particularly for OCD) and ‘active listening’. One article emphasised the importance of technology for facilitating and maintaining a vital ‘support structure’.  In practical terms a number of peer support groups offered advice and information on services, other sources of support, medication and obtaining other basic resources. For example, from China there were two examples of online peer groups exchanging advice on how to obtain their medication. A perinatal befriending service was reported as providing a breast pump to one mother to allow her to express milk so her partner could feed their baby while she was unwell. |
| Creative digital and online approaches | 9 | USA (3), UK (2), France (1), Germany (1), International (1), Mexico (1) | Journalist (6), Person with relevant lived experience (2), Clinician (1), Scientist (1) | Media article (5), Organisational website (2), Blog (1), Specialist health and social care press (1) | Peer and mutual support groups had worked in creative and responsive ways to provide support, advice and connections using online, digital and social media as well as telephone and postal contact. A diverse range of platforms and applications were being utilised, including Twitter, Facebook, Skype, Zoom, texting, webinars, instant messaging, podcasts on coping strategies and WhatsApp groups. There were specific examples of online peer support groups, gaming groups and shared film streaming sessions. |
| Communicating and connecting | 10 | USA (3), UK (2), France (1), Germany (1), International (1), Mexico (1) | Journalist (6), Person with relevant lived experience (2), Clinician (1), Scientist (1) | Media article (5), Organisational website (2), Blog (1), Specialist health and social care press (1) | Communicating and connecting were reported as being important for reducing social isolation during lockdown, maintaining relationships and social connections, enabling individuals to support each other with mental distress and other difficulties, and for socialising and having fun. One individual reported that connections with people who knew their mental health history was ‘lifesaving’. Communication appeared to be happening on a group and individual basis. One individual described connecting with peers as a ‘therapeutic intervention’.  Notably, articles from Italy and Germany mentioned the importance and positive impacts of communicating and connection within inpatient environments during the pandemic. Humour as a means of connecting was mentioned in articles on inpatient environments from the UK and Germany. |
| Family and friends | 9 | UK (4), USA (3), Italy (2), Australia (1) | Journalist (5), Person with relevant lived experience (3), Clinician (2), Policy, professional and charity sector (1) | Media article (5), Organisational website (3), Specialist health and social care press (1), Video/Webinar/Podcast (1) | As also discussed in the Loneliness and Social Isolation section (Table 3) several articles highlighted the role of keeping in touch with family and friends for supporting mental health and alleviating isolation during lockdown. One article emphasised the importance of ‘staying in touch virtually’, which was reported to help keep a routine. Another reported how family and friends could help individuals manage or even prevent relapse by drawing on their experience of what had helped in the past. Family and friends reportedly offered practical support for collecting medication. |
| Helping and contributing | 7 | UK (3), USA (2), Australia (1), Italy (1) | Journalist (3), Clincian (2), Person with relevant lived experience (2), Policy, professionaland charity sector (2) | Media article (3), Organisational website (3), Video/Webinar/Podcast (1) | Mutual aid, peer support and befriending appeared to have positive benefits for those offering the support. Some articles included testimonies from those for whom helping and contributing to lockdown related mental health and other activities was important for their own wellbeing. One article reported a woman finding respite from distress in being able to ‘feel useful’ to others, while articles reported on those who found ‘meaning in little things’ like volunteering, helping parents and supporting the homeless. Another reported that those finding their symptoms were easing in the lockdown felt an increased capacity to help others. |
| Sharing stories and experiences | 4 | Germany (1), Interantional (1), Mexico (1), USA (1) | Journalist (2), Person with relevant lived experience (1), Scientist (1) | Media article (2), Blog (1), Video/Webinar/Podcast (1) | The majority of one-to-one and group peer support consisted of sharing stories and experiences, both managing mental health generally and coping in lockdown and in the wider pandemic crisis. Some articles included mention of sharing coping strategies, stories of positive adaptations and experiences of services. |
| Community support | 2 | Germany (1),UK (1) | Clinciian (1),Person with relevant lived experience (1) | Media article (2) | Very few of the sources we retrieved referred to support provided by neighbours or the wider local community. One UK-based author wrote that their village had set up a valuable Facebook group for those who were isolating and needed home deliveries. A German source reported seeing a revival of community-based mutual aid during the pandemic, for example, offers to watch neighbours’ children. |

**Service impacts**

## **Table 9:** *How has service activity changed? Reports on changes in the early part of the pandemic*

**Total unique sources: 136**

**Theme summary:**

A mixed pattern emerged from these reports, many of them from the very early stages of the pandemic. In secondary mental health services, most reports were of reduced levels of activity, with hospitals, emergency departments and community services initially reporting fewer & Emergency (A&E) fewer admissions, presentations and referrals respectively. Of note, these were generally reporting from individual clinicians or managers (sometimes reported to journalists) rather than official data sources. Discussions of this phenomenon suggested that service users avoiding hospitals due to fear of infection or trying to avoid burdening services, and services focused mainly on emergencies only may contribute to this. Meanwhile there were many reports of large increases in people contacting hotlines focused on mental health or suicide prevention round the world, as well as in people accessing digital care.

| *Sub-theme* | *Number of sources* | *Countries* | *Author types* | *Source types* | *Content* |
| --- | --- | --- | --- | --- | --- |
| Helpline | 61 | USA (16), UK (15), Germany (13), International (6), Canada (3), France (2), Switzerland (2), Australia (1), Belgium (1), India (1),Spain (1), | Journalist (46), Policy, professional and charity sector (8), Clinician (4), Persons with relevant lived experiencd(4), Scietists (3), Unknown (3) | Media article (44), Organisational website (7), Specialist health and social care press (4), Video/Webinar/Podcast (3), Blog (1), Journal article (1), Policy body (1) | A large majority of helplines, with any focus and in any countries, reported substantial increases in numbers of calls, sustained after the pandemic crisis on onset, with reports from around third more calls to twice as many as usual seeking help. For example, a suicide prevention hotline in Portland, Oregon, reported a 41% call in calls to its suicide hotline after a “state of emergency” was declined, and in Germany a suicide helpline reported a doubling in demand. |
| Hospital | 20 | Italy (10), France (5), UK (2), USA (2), Spain (1) | Journalist (11), Clincian (7), Policy, professional and charity sector (2), Scientist (1), Unknown (1) | Media article (10), Specialist health and social care press (5), Organisational website (4), Journal article (1) | Most sources reported decreases in admissions to psychiatric hospitals in the very early phases of the pandemic period, ranging from 15% to a 66% reduction in the number of hospital beds occupied. Potential explanations included staff deliberately managing people at home as far as possible; personal and staff efforts to burden the hospital as little as possible, concerns about infection in hospital among service users, and concerns among staff of risk of infection for service users.    However, five reports (four from Italy reported increased admissions to hospital, with a further Italian article reporting an initial trend in fewer admissions during the first few weeks of the crisis, then followed by a marked increase in compulsory hospitalisations after a few weeks of lockdown. |
| Digital | 15 | USA (10), UK (3), Germany (1), International (1), | Journalist (11), Policy, professional and charity sector (2), Unknown (2) | Media article (13), Organisational website (2) | 15 sources report increased access to digital care, either through accessing apps, online therapy or website support, however reports are limited to three countries. For example, IESO, an online Cognitive Behavioural Therapy service in the UK, described a 40% increase in referrals early in the pandemic crisis. |
| Community | 15 | UK (7), International (2), Italy (2), Australia (1), Canada (1), Germany (1), USA (1) | Journalist (9), Policy, professional and charity sector (3), Clinician (2), Person with relevant lived experience (1), Scientist (1), Unknown (1) | Media article (6), Organisational website (5), Specialist health and social care press (3), Policy body (1) | Reports regarding activity in community services were limited and mixed. In mainstream community mental health services there seemed often to be reports of falls in activity: for example a 50% in Child & Adolescent Mental Health Service (CAMHS) referrals in Birmingham since the outbreak and a 30% reduction in Improving Access to Psychological Therapy (IAPT) referrals (for brief talking therapy) in East London & Yorkshire.  However, referrals were reported to have risen for voluntary sector mental health services (in the UK and Australia) |
| Accident & Emergency  (A&E) | 9 | France (4), Spain (2), Australia (1), Italy (1), UK (1) | Journalist (5), Clincian (3), Scientist (3) | Media article (4), Journal article (3), Specialist health and social care press (2) | Six sources reported declines in the number of presentations to Psychiatric A&E, with the number of patients attending to a psychiatric Emergency Department in Madrid reduced by 75%. The most common explanation given for declines in A&E attendance was of patients worried about contracting coronavirus.  In Paris a psychiatric emergency department received around 33% more patients presenting five weeks after the start of lockdown, and one NHS Trust report that they have experienced an increase in individuals presenting at A&E with mental distress. |
| Medication | 4 | USA (3), International (1) | Journalist (3), Clincian (1), Person with relevant lived experience (1) | Media article (4) | Four sources report on the evidence for increased demand for psychiatric prescriptions in the early phase of the pandemic: according to a national US pharmacy, prescriptions for antidepressants, anxiety and insomnia medication has increased by 21% between 16.02.20 to 15.03.20. |

**Service challenges and adaptations in inpatient and residential settings**

## **Table 10:** *Challenges in inpatient and residential settings during the pandemic*

**Total unique sources: 190**

**Theme summary:**

Inpatient and residential settings (such as hostels and supported housing for people with mental health problems) are the settings in which infection control challenges are at their most acute, given high risks if the virus begins to spread. Specific challenges in infection control in these settings include difficulties for very unwell or distressed patients in following guidance, and ward layouts that impede physical distancing. Depleted resources can make this challenge harder to address, with discussions of physical healthcare currently having still greater priority over mental health care, with staff and physical resources redeployed and patients having to be turned away or discharged early due to lack of capacity. Workforce depletion results from illness, caring responsibilities, concern about infection and redeployment. Sources describe guidance about how to operate during the pandemic as limited, and infection control measures making delivering good quality care very challenging in this setting, as are face-to-face consultations and access to group-based therapeutic activities, and in some settings inpatients are largely confined to their rooms. Psychiatric hospitals/units often continue to treat their patients if they contract COVID-19 unless they need a very high level of medical intervention, and significant challenges are described in accessing appropriate equipment and expertise for this physical healthcare. Concern about limited access to Personal Protective Equipment (PPE) was prominent across the sources from a variety of countries, along with the challenges of physical distancing given the community structure and physical layout of many psychiatric wards.

| *Sub-theme* | *Number of sources* | *Countries* | *Author types* | *Source types* | *Content* |
| --- | --- | --- | --- | --- | --- |
| Infection control in psychiatric units/residential settings | 90 | France (21), UK (15), Italy (13), USA (10), Germany (7), Spain (4), Belgium (3), China (3), Switzerland (2), Canada (1), Colombia (1), Ghana (1), Singapore (1) | Journalist (49), Clinician (26), Scientist (13), Person with relevant lived experience (6), Policy, professional and chariety sector (6), Unknown (4) | Media article (46), Specialist health and social care press (17), Organisational website (13), Journal article (9), Video/Webinar/Podcast (3), Policy body (2) | A number of sources describe specific issues with infection control. Efforts are often made to minimise the risk of infection by introducing enhanced PPE and cleaning policies and reducing the number of patients on wards, however sources from several countries report a lack of PPE available to staff and patients, sometimes suggesting that mental health settings are prioritised less than physical health care. Some also report difficulties in accessing virus testing for their patients leading to challenges in admission and treatment.  The difficulty of maintaining physical distancing within these settings is also highlighted; some patients are unwell enough that they may not fully understand the need for social distancing and protective equipment or are unwilling to comply with new safety measures. Ward layouts also sometimes impede distancing. Units often create COVID-19 and non-COVID-19 wards so that they can continue to treat patients who have contracted the virus whilst trying to minimise infection across the unit. |
| Inpatient restrictions | 41 | France (11), UK (7), Germany (5), International (4), USA (4), Italy (3), Belgium (2), China (2), Canada (1), Singapore (1), Switzerland (1) | Journalist (17), Clinican (16), Scientist (7), Unknown (4), Person with relevant lived experience (3), Policy, professional and charity sector (3) | Media article (19), Organisational website (7), Journal article (6), Specialist health and social care press (5), Video/Webinar/Podcast (2), Blog (1), Policy body (1) | In order to control the spread of the virus, hospitals have put restrictions in place with considerable impact on patients. Wards have become cut off from the community, so visitors to inpatients are often not allowed, and leave for inpatients has been cancelled.  A frequently reported practice is for new inpatients to be quarantined in their rooms for 14 days, and for existing inpatients to have less access to communal spaces and to spend more time confined to their rooms. Often group activities such as group therapy have been cancelled and consultations/individual support may happen through phone or video calls. Sources emphasise the impoverishment of inpatient care, and the potentially damaging impact of this lack of engagement and social interaction. |
| De-prioritisation | 43 | France (14), UK (9), Italy (7), USA (4), Germany (2), International (2), Belgium (1), Canada (1), China (1), Ghana (1), Spain (1) | Journalist (21), Clincian (11), Scientist (10), Policy, professional and charity sector (5) Person with relevant lived experience (4), Unknown (3) | Media article (19), Organisational website (9), Specialist health and social care press (7), Journal article (5), Policy body (2), Video/Webinar/Podcast (1) | Although inpatient units generally remain open in some capacity, a view is expressed in many sources that they units are being treated as less of a priority than physical healthcare units. Issued guidance is often focused on physical healthcare rather than psychiatric care, leaving units without clear policies as to how to operate. Staff may be redeployed to other units and equipment such as PPE is also primarily given to other hospital units. Supported housing and community residential units appear to be still less of a priority, despite the considerable potential risks related to infection in these settings.  Many sources report that the amount of psychiatric beds available has been reduced, as psychiatric wards have been re-designated as COVID-19 wards. Given reduced capacity, thresholds for admission have been raised so that only the most acute cases are considered for admission and patients are being released earlier than originally planned to free up space. |
| Staffing and expertise available | 45 | UK (16), France (8), Italy (6), USA (5), Spain (3), Belgium (2), China (2), Germany (1), International (1), Singapore (1) | Journalist (23), Clinician (13), Scientist (8), Policy, professional and charity sector (4), Unknown (3), Person with relevant lived experience (1) | Media article (19), Organisational website (9), Specialist health and social care press (6), Journal article (5), Policy body (3), Video/Webinar/Podcast (2), Blog (1) | There are also general staffing shortages as staff have to take time off due to being infected with coronavirus or having caring responsibilities. There are some reports of remaining staff having to work significantly longer hours than usual or take on more shifts to cover shortages. Some hospitals have restructured their staffing, either in response to shortages or to minimise the spread of the virus through staff.    Sources also state that staff trained for psychiatric care are not experienced in physical health care, so looking after COVID-19 patients on their wards is challenging and requires additional support. Concerns are expressed in several sources, including in a public letter by a large group of French psychiatrists, that pressure to treat people with mental illnesses and COVID-19 as far as possible in psychiatric units may be harmful and inequitable because of lack of expertise and equipment. There is concern about the additional stress and emotional burden being placed on staff, with some reporting that they now feel unsafe at work. |

## **Table 11:** *Service adaptations and innovations in inpatient and residential settings*

**Total unique sources: 87**

**Theme summary:**

For inpatient and residential services, the most commonly discussed adaptation and innovation to services was creation of COVID-19 specific units for psychiatric patients with confirmed or suspected illness. In some settings, liaison services with expertise in physical health supported these in adapted wards or dedicated areas, with safety protocols in place if the patient required a transfer to intensive care. Several sources described using technology to address the impoverishment of inpatient settings due to infection control measures, introducing new technological facilities to enable patients to have remote contact with to maintain therapy, and with families to maintain contact whilst visitations were restricted. In some services, ways of maintaining provision such as group therapy sessions and visitations/outings under certain circumstances with protocols involving use of PPE and physical distancing. Further infection control measures described in inpatient settings included schedules at mealtimes, reductions in numbers of patients on wards and numbers of people in group treatment. Protocols related to admission and discharge have also been adapted, with some early discharges and a few sources discussing initiatives to increase home treatment in order to avoid hospital admissions where possible. Discussions of managing infection control in residential settings were few.

| *Sub-theme* | *Number of sources* | *Countries* | *Author types* | *Source types* | *Content* |
| --- | --- | --- | --- | --- | --- |
| COVID-19 specific units | 27 | France (16), Italy (3), China (2), UK (2), International (1), USA (1), Spain (1), Germany (1) | Journalist (13), Clinician (10), Scientist (6), Policy, professional and charity sector (2), Unknown (2) | Media article (14), Journal article (5), Organisational website (4), Specialist health and social care press (4) | COVID-19 specific units have been introduced in many settings to meet need in the current situation. This involves a specific unit for psychiatric patients with COVID-19, or suspected COVID-19. Initiatives were described in some settings to improve the care on these units through additional liaison with physical health services and professionals. Development of protocols for safe transfer from these settings to general hospitals and intensive care units is described. However, as in the preceding section on challenges in inpatient units, concerns have been raised regarding quality of physical healthcare that can be delivered within psychiatric hospitals for people with mental health problems and COVID-19 infection. Some sources discuss possible mental health impacts of novel COVID-19 therapies, such as the potential for hydroxychloroquine to trigger psychosis. |
| Technology | 24 | France (6), Italy (4), USA (4), UK (3), Germany (3), Spain (2), International (2) | Journalist (12), Clinician (10), Scientist (6), Policy, professional and charity sector (2), Person with relevant lived experience (1) | Media article (10), Specialist health and social care press (7), Organisational website (4), Journal article (3) | Some initiatives involve enhancing availability of technology on wards to address the isolation and limited therapeutic programme that may result from infection control measures. Technology is described as being used to facilitate remote contact between services and patients, and also between patients and their families. This includes remote contact over the phone and video conferencing software. One source described how these measures have been positively received by patients. Other sources describe debates about the safety and appropriateness of additional availability of technology-enabled communication software. |
| Links with community | 16 | France (7), Italy (4), UK (3), Spain (1), Germany (1) | Journalist (9), Clinician (6), Scientist (3), Unknown (1) | Media article (8), Specialist health and social care press (3), Journal article (2), Organisational website (2), Video/Webinar/Podcast (1) | Some sources discuss admissions and discharges during the pandemic period. Hospital admissions continue in most settings, but four sources described initiatives to increase capacity for intensive home treatment in order to free up hospital beds and avoid exposing patients to risks of infection in wards. Early discharge policies are described elsewhere, discharging patients to the community where suitable, and supporting patients at home. Given the pressure on health services and the ethical requirement to avoid exposing patients to infection if at all possible, sources discussed reserving inpatient admission to situations of high risk in which it appeared unavoidable. |
| Initiatives to maintain physical distancing on wards | 15 | France (4), USA (4), Italy (3), Germany (1), Belgium (1), Switzerland (1), China (1) | Journalist (10), Clinician (5), Scientist (4) | Media article (9), Journal article (3), Specialist health and social care press (2), Organisational website (1) | Physical distancing protocols have been developed to try to protect staff and patients during the coronavirus pandemic. These include staggering mealtimes, reducing numbers of inpatients on each unit, and limits on numbers in communal areas. In some settings, new inpatients are placed in quarantine in their rooms for 14 days at the beginning of their stays. |
| Services maintained | 12 | France (4), UK (2), Italy (2), Germany (2), Switzerland (1), Spain (1) | Clinician (5), Journalist (5), Scientist (3), Person with relevant lived experience (1), Policy, professional and charity sector (1) | Media article (5), Journal article (3), Specialist health and social care press (2), Organisational website (1), Video/Webinar/Podcast (1) | Sources describe how in some inpatient services, face to face contact and some group activities are still being provided with staff and patients following hygiene and social distancing protocols by wearing PPE e.g. Child & Adolescent Mental Health Services, eating disorder units. Protocols for allowing physically distanced family contact are described in other settings. |
| Introducing Personal Protective Equipment | 11 | France (5), Italy (3), International (1), USA (1), UK (1) | Journalist (6), Clinician (3), Policy, professional and charity sector (2), Scientist (2) | Media article (6), Journal article (2), Organisational website (2), Specialist health and social care press (1) | Several sources discuss use of PPE onwards, and the new procedures and measures required to maintain hygiene, including hygienic facilities for putting on and disposing of PPE, and processes for disinfecting rooms. Some sources described staff resourcefulness in obtaining supplies in the face of shortages, for example by obtaining disposable gowns from a catering company or making masks at home. |

**Service challenges and adaptations in community settings**

## **Table 12:** *Challenges in community settings*

**Total unique sources: 81**

**Theme summary:**

Significant and varied challenges were identified for community mental health services. The primary pressures were from the need to severely restrict face-to-face contact, to enforce infection control measures where such contact is still occurring, and in some cases to free up staff for redeployment to inpatient or general medical settings. A range of services have reduced their capacity or closed entirely. Many sources described reduced workforce capacity, commonly owing to staff sickness or redeployment to other services. Understandably staff have high levels of distress and anxiety at this time. New ways of working such as via remote technologies have been helpful but can involve significant challenges. Not all service users have access to phones, computers or the internet and appropriate space to conduct private calls with care co-ordinators. The wearing of PPE and the nature of video and telephone consultations additionally bring challenges for staff and service users dynamics and relationships. As has been a consistent issue across the whole health sector, inadequate PPE and a lack of access to coronavirus tests was frequently cited by sources. However, low PPE supplies were considered to be even more of an issue in mental health settings compared to physical health settings, and still more in the Voluntary Community and Social Enterprise (VCSE) sector. The VCSE sector has also been negatively impacted by the loss of money due to fundraising events being cancelled.

| *Sub-theme* | *Number of sources* | *Countries* | *Author types* | *Source type* | *Content* |
| --- | --- | --- | --- | --- | --- |
| Disruption to services | 28 | UK (9), Italy (8), Germany (5), France (2), USA (2), Canada (1), International (1) | Journalist (11), Clinician (8), Policy, professional and charity sector (8), Unknown (3), Person with relevant lived experience (1), Scientist (1) | Organisational website (13), Media article (9), Specialist health and social care press (3), Journal article (2), Video/Webinar/Podcast (1) | Sources documented community services reducing and limiting their service, closing it entirely or being only accessible for emergency cases. Some services are no longer taking new referrals.  Settings such as day centres and community centres where service users mingle are particularly likely to have closed. There were discussions, for example in Italy and the UK, of considerable variation in response, with some areas closing down all but urgent care services and others placing a high priority on maintaining service provision as much as possible within national infection control requirements. |
| Workforce issues | 26 | UK (12), USA (5), Germany (3), Italy (3), Canada (1), Singapore (1), Switzerland (1) | Journalist (14), Policy, professional and charity sector (5), Clinician (3), Unknown (3), Person with relevant lived experience (2), Scientist (1) | Media article (12), Organisational website (9), Specialist health and social care press (2), Blog (1), Journal article (1), Video/Webinar/Podcast (1 | Seventeen sources stated that community services were understaffed mainly owing to staff illness or redeployment to other mental and physical health services, sometimes with immediate impacts on mental health service provision. Some providers, such as in the NHS, described the return of former staff and those in other employment, such as universities, to help address this situation. In some countries with insurance-based health system, the ability to continue services remotely was limited because some insurance coverage does not include tele-health. |
| Inadequate PPE | 19 | Italy (7), UK (5), USA (3), France (2), Germany (2) | Journalist (6), Policy, professional and charity sector (5), Clinician (4), Unknown (4) | Organisational website (9), Media article (5), Specialist health and social care press (3), Policy body (1), Video/Webinar/Podcast (1) | Sources detailed how in a range of different community mental health services there was a lack of adequate PPE for staff and for patients seen in-person, ultimately in some cases resulting in service closure. Mental health services in the community were viewed as being “low priority” for PPE supplies by authorities. UK Voluntary Community and Social Enterprise (VCSE) services appear to be the most affected by low PPE levels, with the majority of stocks allocated to the NHS first. A source from Italy reports difficulties with encouraging mask wearing by patients. A UK survey by the Royal College of Psychiatrists found that, in the UK overall, 23% of psychiatrists did not have access to correct PPE. |
| Impediments to face to face treatment | 13 | Germany (6), Italy (3), USA (2), International (1), UK (1) | Journalist (6), Clinician (5), Unknown (2) | Media article (7), Organisational website (3), Journal article (2), Video/Webinar/Podcast (1) | Where meetings do take place face-to-face, therapeutic relations between staff and service users are reportedly impeded by PPE limiting communication and the ability to gain a full impression of someone. There are difficulties in administering eating disorder services as they often involve weighing service users in person to track their weight. |
| Challenges with remote care | 12 | Germany (5),UK (2), USA (2), France (1), International (1), Italy (1) | Journalist (9), Clinician (2), Policy, professional and charity sector (1), Unknown (1) | Media article (8), Specialist health and social care press (2), Organisational website (1), Policy body (1) | Issues raised with delivering mental health care via phone or video call include difficulties establishing a therapeutic relationship and the impact on the dynamics between service user and clinician, difficulties for staff in working from home with sufficient equipment and privacy, and a lack of relevant training on teleconsultations. Furthermore, many service users may not have the access to technology for phone or video calls, and if they do, they may not have a suitable place in their house to discuss private matters with mental health staff away from co-habiting family. |
| Workforce challenges | 8 | Italy (3), UK (2), USA (2), Australia (1) | Clinician (3), Policy, professional and charity sector (2), Scientist (2), Journalist (1), Unknown (1) | Specialist health and social care press (3), Media article (2), Organisational website (2),Policy body (1) | Stress and worry about contracting the virus for those working face-to-face in the community, and impacts on personal lives from school closures were among the workforce challenges impacting on services (workforce well-being was not, however, in the scope of our review) |
| Financial challenges | 5 | Italy (2), International (1), Switzerland (1), UK (1) | Person with relevant lived experience (2), Policy, professional and charity sector (2), Journalist (1) | Media article (2), Organisational website (2), Specialist health and social care press (1) | VSCE services have experienced a significant drop in funding due to cancelling various fundraising initiatives. Meanwhile, volunteers may have been diverted elsewhere. |
| Lack of access to tests | 3 | UK (3) | Unknown (2), Policy, professional and charity sector (1) | Organisational website (3) | Three UK sources described a lack of coronavirus testing provision to mental health care staff, with VCSE services again experiencing particularly low provision of tests.  A UK survey by the Royal College of Psychiatrists found that only half of psychiatrists were able to access tests for themselves (51%) or their clients (54%), and only 30% were able to access family tests. |

## **Table 13:** *Service adaptation and innovations in community settings*

**Total unique sources: 417**

**Theme summary:**

Services across the globe were innovative in adapting their community care to ensure continued access during the pandemic. Technology is now a prominent treatment modality in services, allowing care to continue both through video call therapy sessions, and phone lines to try to ensure access continues for all. Greater use of digital tools, such as apps and websites is also described. Proactive redesigning of protocols and services has also taken place to try to continue an adequate level of support for those who most need it. Reports from multiple sources emphasise the necessity of retaining some level of support for people with mental health problems, and this is reflected in efforts to adapt care as well as funding provision and insurance eligibility alterations.

| *Sub-theme* | *Number of sources* | *Countries* | *Author types* | *Source types* | *Content* |
| --- | --- | --- | --- | --- | --- |
| The shift to telehealth | 273 | UK (65), France (42), Italy (41), USA (41), Germany (30), International (17), China (10), Spain (8), Canada (3), Switzerland (3), Australia (2), Belgium (2), Colombia (2), Singapore (2), Austria (1), Ghana (1), India (1), Mexico (1), Peru (1) | Journalist (124), Clinician (76), Policy, professional and charity body (37), Scientist (36), Unknown (17), Person with relevant lived experience (13) | Media article (115), Organisational website (64), Specialist health and social care press (46), Journal article (26), Video/Webinar/Podcast (11), Policy body (8), Blog (3) | The shift to telehealth was a major theme emerging from the literature. For example, many sources (228) from a variety of countries discussed how services have maintained treatment through providing interventions and consultations via video call or telephone.  Similarly, 22 sources mentioned that peer support has also adapted to the situation, continuing support via online platforms, e.g. Facebook groups, chat forums, and use of zoom meetings for one-to-one chats. Many highlighted the importance of maintaining peer support channels to reduce loneliness.  A number of benefits of telemedicine innovations were discussed. Some felt they allowed increased access to services, particularly for those who may find face-to-face intervention an anxiety-provoking experience.  Some clinicians reported benefits from additional insights gained from seeing patients’ living spaces.  However, 65 sources described pitfalls of the shift to telehealth, and a common opinion was that telehealth could not adequately replace face-to-face therapy long-term, as it can negatively affect the therapeutic relationship through the distance it creates.    Furthermore, there were concerns that the use of technology for telehealth may highlight disparities, excluding those who do not own, or are less able to use technology required, disproportionately affecting groups such as older people, people with intellectual disabilities and those living in poverty.  Many also felt that telehealth appointments could be distressing, as they felt like an invasion of privacy, or because patients lacked the privacy to discuss topics with their clinician. |
| Increasing access | 220 | UK (70), USA (39), Germany (29), France (24), Italy (18), International (10), Australia (6), China (6), Canada (5), Switzerland (4), Greece (2), Austria (1), Belgium (1), India (1), Ireland (1), Peru (1), Singapore (1), Spain (1) | Journalist (118), Policy, professional and charity sector (44), Clinician (33), Scientist (21), Person with relevant lived experience (9), Unknown (17) | Media article (108), Organisational website (60), Specialist health and social care press (25), Journal article (10), Policy body (9), Video/Webinar/Podcast (8) | Two-hundred and twenty sources discussed innovations and adaptions put in place by services to increase access to mental health care during the coronavirus pandemic.  Many described how services were targeting those service users most in need to ensure the crisis didn’t prevent them getting support, and face-to-face services were retained in many services for those patients who needed them most, or for emergency cases.  Additionally, some sources described innovations to increase capacity of services to meet the growing demand, for example the conversion of closed hospitals, opening temporary care centres and moving staff to mental health departments. The extension or opening of 24/7 crisis lines was frequently mentioned, to ensure those who need support can access it somehow.  Also commonly discussed was the development of apps and websites to allow increased access to support amid growing demand. These were particularly used as self-help material provision, or a means of connecting people to relevant services for their needs. Some apps even provided low intensity support or formed a basis of reaching otherwise unreachable people in crisis. In some cases, services sought to ensure patients had access to WIFI or a phone during the crisis to ensure continuing support.  Finally, twelve sources discussed increases in funding for mental health services to allow innovations to be implemented, and similarly, relaxing of insurance rules and regulations surrounding therapist working hours via remote methods were frequently referred to, as at the onset of the crisis, some health coverage in insurance-based systems did not include tele-health. . |
| Reorganisation of services | 83 | UK (20), Italy (12), France (11), USA (10), Germany (9), China (6), International (6), Spain (3), Belgium (2), Australia (1), Canada (1), Colombia(1), Ghana (1) | Journalist (33), Clinician (22), Policy, professional and charity sector (19), Scientist (14), Person with relevant lived experience (5), Unknown (5) | Media article (28), Organisational website (23), Specialist health and social care press (12), Journal article (11), Policy body (4), Video/Webinar/Podcast (3),  Blog (2) | There are many descriptions of adaptations to the crisis through redesigning services and treatment protocols. Some ensured social distancing guidelines could be followed in order to allow face-to-face appointments to continue: groups were made smaller, hygiene procedures implemented and team mixing reduced. Scheduling was also adapted to reduce waiting times in enclosed spaces. Sources describe introduction of systems for delivering medication to patients’ homes and provision of extended prescriptions. Some sources describe additional home visits to avoid patients having to come to hospital sites. Some describe adaptation of treatment protocols to include COVID-19 discussion or make them more relevant to the current situation in a few cases, e.g. through addition of bereavement counselling or specific discussion of how to cope with COVID-19 symptoms in therapy.  Other organisational adaptations described in some countries include increased coordination between departments to meet demand, for example through professionals travelling from less affected areas of countries, or the creation of multidisciplinary task forces. In 12 sources, it was mentioned that staff were provided with additional online training, both to help deal with the adaptions in services and the training of front-line workers in psychological first aid. |
| Recognising mental health services as essential | 19 | UK (9), Germany (4), Canada (3), Italy (2), USA (1) | Journalist (11), Clinician (4), Policy, professional and charity sector (4) | Media article (11), Organisational website (5), Policy body (2), Video/Webinar/Podcast (1) | Several sources emphasise the importance of recognising mental health care as an essential service. There were concerns that the extent of closures, at least in some areas, may be disproportionate to the crisis and to the severe potential consequences of discontinuing support. |

**Ethical challenges**

## **Table 14:** *Ethical challenges and challenges to values:* *Inequalities, denial of treatment or provision of inadequate treatment, challenges associated with compulsory admission*

**Total unique sources: 58**

**Theme summary:**

Several issues were raised around ethical challenges in maintaining professional values and human rights in the context of the pandemic. The issues raised centre in particular - although not exclusively - on inpatient psychiatric settings. Some sources argue that access to physical health care (for COVID-19) is inequitable for mental health service users, and that they may receive poorer quality health care, due to stigma and decisions to try to treat them within psychiatric units rather than admit them to general hospitals. There are also instances of concerns expressed that mental health care has become - or has the potential to become - less ethical during the pandemic. These concerns include beliefs that medication doses and the use of sedation may have increased, that coercive and restrictive practices which impact rights and freedoms are on the rise, and that there has been an increase in hierarchical power exerted over service users within mental health services. Additionally, there are concerns that legislative changes introduced during the pandemic, such as the Coronavirus Act (2020) in the UK, curtail service users’ rights (for example by enabling involuntary inpatient admissions to be made with agreement by fewer healthcare professionals, extending time limits on detention, and facilitating the use of treatment without consent) while, at the same time, access to legal representation and advocacy may be more difficult.

| *Sub-theme* | *Number of sources* | *Countries* | *Author types* | *Source types* | *Content* |
| --- | --- | --- | --- | --- | --- |
| Inequitable access to physical health care | 21 | France (9), Italy (4), UK (3), International (2), Belgium (1), Germany (1), Ghana (1), | Clinician (8), Journalist (5), Scientist (4), Person with relevant lived experience (3), Policy, professional and charity sector (3) | Media article (9), Organisational website (6), Specialist health and social care press (4), Blog (1), Policy body (1) | Concerns are raised that access to physical health care (for COVID-19) is inequitable for mental health service users. This theme is seen particularly among sources from France, where a group of psychiatrists had written expressing serious concerns that psychiatric patients with COVID-19 are receiving inadequate and inequitable care due to policies of treating them in psychiatric rather than general hospitals for as long as possible. There are also concerns that mental health patients may be deprioritised for treatment, or receive poorer quality treatment, due to stigma, and that general hospitals do not cater well for those with mental health difficulties. |
| Less ethical care, especially in inpatient units | 18 | France (7), UK (5), International (2), Canada (1), Germany (1), Italy (1), USA (1) | Clinician (6), Journalist (5), Person with relevant lived experience (5), Scientist (2), Policy, professional and charity sector (1), Unknown (1)0 | Media article (8), Organisational website (6), Specialist health and social care press (2), Blog (1), Policy body (1) | There are concerns about mental health care becoming (or having the potential to become) less ethical as a result of the pandemic, particularly in inpatient psychiatric settings.  Issues cited include beliefs that medication doses and sedation have been increased; that there has been an increase in hierarchical power exerted by clinicians over service users; that use of restraint and coercive practices has risen; that confining service users to their rooms and denying them access to family members and carers impinges on freedoms; and that information provision may now be poorer particularly for service users with social or communication difficulties. Ethical challenges where compulsory admission places service users at greater risk of infection are discussed. |
| Legislative changes curtailing rights | 18 | UK (15), France (1), Germany (1), International (1) | Policy, professional and charity sector (9), Person with relevant lived experience (5), Scientist (2), Clinician (1), Journalist (1), Unknown (1) | Organisational website (9), Specialist health and social care press (4), Media article (2), Policy body (2), Video/Webinar/Podcast (1) | Concerns that legislative changes have been introduced during the pandemic were highlighted, which risk diminishing service users’ rights. There is a particular emphasis on the Coronavirus Act 2020 in the UK: although at the time of writing its provisions had not come into force, there were fears that if enacted, it would reduce rights and safeguards by allowing involuntary inpatient admissions to be made with agreement by fewer healthcare professionals, with extended time limits and fewer restrictions on treatment without consent. With a number of so-called ‘places of safety’ also being closed, there are also concerns that more people may be held in police cells |
| Difficulties accessing advocacy/legal support | 4 | UK (3), France (1) | Policy, professional and charity sector (3), Clinician (1) | Organisational website (2), Blog (1), Policy body (1) | There are some concerns that access has become more difficult to advocates or legal representatives for service users during the pandemic. |

**Expectations and concerns for future**

## **Table 15:** *What do people anticipate, hope for, or fear in the future?*

**Theme summary:**

Most sources discussing the future anticipated an increase in demand as one of the major future challenges that mental health services will likely have to tackle worldwide, as many expect the pandemic to have a delayed impact on service users’ mental health. The main concerns focused around shortage of beds and the appropriate management of symptoms and relapses and led to the planning of new strategies involving the provision of ad hoc resources and preventive plans. Socio-economic challenges were anticipated particularly in countries already suffering from a financial crisis; however, these were expected in other countries as well, with charities being especially at risk. People with mental health conditions were expected to experience even greater psychological distress, and some anticipated greater risk of suicide, although the need to avoid assumptions about this was also emphasised.  Many sources also suggested that continuing to make greater use of technology platforms for delivery of care is desirable. Some countries described the pandemic as an opportunity to concentrate more resources on mental health care and to solve existing problems. Finally, a few sources emphasised the need for appropriate protective equipment and monitoring systems in order to avoid new outbreaks.

| *Sub-theme* | *Number of sources* | *Countries* | *Author types* | *Source types* | *Content* |
| --- | --- | --- | --- | --- | --- |
| Increased demand and new strategies | 63 | UK (18), USA (12), France (10), Italy (8), International (4), China (3), Germany (3), Spain (3), Canada (1), Dominican Republic (1) | Journalist (30), Clinician (23), Scientist (9), Policy, professional and charity sector (8), Person with relevant lived experience (2), Unknown (1) | Media article (28), Organisational website (12), Specialist health and social care press (11), Journal article (7), Policy body (2), Video/Webinar/Podcast (2), Blog (1) | Many people, including mental health staff, anticipated that mental health services and psychiatric hospitals worldwide will experience a significant increase in demand and admissions due to the consequences of the pandemic on people’s mental health, including effects of trauma and bereavement. Of note, one clinician argued for greater reserve in making predictions, as the situation is unprecedented, and vulnerable people sometimes prove more resilient than expected.  Some seemed to be particularly concerned about the shortage of beds and general mental health services capacity, while others focused on maintaining good care principles when managing symptoms, relapses and suicide risk.  Strategies to prepare for this challenge are advocated, such as preparation of emergency and continuity-of-operations plans should be, and investment in mental health services in general, and especially in services crisis and assessment units, and suicide prevention strategies. |
| Socio-economic challenges | 33 | Italy (12), UK (9), USA (7), Germany (2), Australia (1), Austria (1), Spain (1) | Clinician (11), Journalist (9), Policy, professional and charity sector (8), Scientist (3), Person with relevant lived experience (2), Unknown (1) | Organisational website (11), Media article (10), Specialist health and social care press (9), Journal article (2), Video/Webinar/Podcast (1) | Many sources predict that the pandemic will have a significant social and financial impact on both mental health services and service users, which in turn will increase psychological distress.  Existing under-resourcing of services intensifies these concerns. Some sources highlighted that charities will particularly suffer from a lack of funding and fundraising unless they receive prompt support. There are concerns that some services that have closed during the pandemic may not re-open.    Some people were concerned about the changes in the legislation which would deprioritise mental health care and limit mental health support in general. Re-deployment of mental health staff was expected to limit the time and resources once allocated to mental health services and their users.  One source reported that service users will not be able to take advantage of some digital services because they cannot afford it, particularly those who are unemployed. |
| Use of technology | 28 | International (6), Italy (6), USA (6), UK (5), Spain (2), Australia (1), Austria (1), Canada (1), | Journalist (14), Clinician (9), Scientist (5), Unknown (2) | Media article (12), Journal article (9), Specialist health and social care press (6), Organisational website (1) | Many sources reported that delivery of care has now been made possible via technology platforms in most countries and it is widely accepted in opposition to the past. Examples included telemedicine, virtual emergency treatments, and more personalised treatment plans.  There was some focus on the positive aspects of using them in mental health care, particularly in countries where mental health resources are scarce. Some strongly recommended keeping on using these virtual tools even when the pandemic is over to accommodate the needs, although limitations are also recognised as in the preceding table, and there are populations such as the homeless who are unlikely to be reached. |
| Challenges for people with mental health problems | 26 | UK (7), Italy (6), USA (6), France (2), Germany (2), International (2), Canada (1) | Journalist (15), Clinician (8), Policy, professional and charity sector (3), Scientist (3), Person with relevant lived experience (2) | Media article (17), Organisational website (5), Specialist health and social care press (2), Blog (1), Policy body (1) | Some sources predict that, following the pandemic, people with pre-existing mental health problems will struggle even more to regain some sort of balance, and relapses are expected. Among the causes, they reported lack of socialisation, care, and work groups and projects.  One clinician highlighted the presence of persisting stigma towards psychiatric patients as a further challenge.  Some specific conditions were also mentioned: It was perceived that people with anxiety disorders, OCD, eating disorders, psychosis, substance use disorders and gambling may be especially in need of support following the crisis, and may have either not had access to or not engaged with help they may need. |
| Positive considerations and hopes | 11 | Italy (5), France (3), UK (2), Spain (1) | Clinician (8), Journalist (3), Scientist (2) | Specialist health and social care press (4), Media article (4), Journal article (2), Organisational website (1) | In order to face the current challenges, some sources described changes that have been implemented. Some people reported that consultations by phone are working and patients are reacting well during the emergency. They reported believing that delivery of resources and hospitalisation at home could serve as a good alternative to admission to a psychiatric hospital unit. In Scotland, funding has been released by the government to help the expansion of counselling services.  Some sources reported that, in countries such as Italy and France, there is hope that the government will pay more attention to the mental health care system as a fundamental resource, and clinicians also hope that this situation will lead to an upgrade in the way therapies are conducted with positive developments and new forms of solidarity, for example between clinicians and service users in seeking to address problems and inequities that have been highly visible during the pandemic. |
| New outbreak and virus infection | 4 | Italy (2), Belgium (1), USA (1) | Clinician (2), Journalist (2) | Media article (3), Specialist health and social care press (1) | A few discussions were also found of the need to prepare for new peaks in COVID-19, infection, and of challenges due to the uncertainty about how the pandemic will progress and when it may end. |

# **Section 2 - Search Strategy**

**SEARCH STRATEGY**

**Published literature databases**

PsychINFO

| 1 | ((cov$ adj4 "2019") or covid-19).mp. [mp=title, abstract, heading word, table of contents, key concepts, original title, tests & measures, mesh] | 20 |
| --- | --- | --- |
| 2 | covid-19.mp. | 7 |
| 3 | (SARS cov 2 or SARS-cov-2).mp. [mp=title, abstract, heading word, table of contents, key concepts, original title, tests & measures, mesh] | 0 |
| 4 | coronaviru$.mp. [mp=title, abstract, heading word, table of contents, key concepts, original title, tests & measures, mesh] | 87 |
| 5 | 2019-ncov.mp. | 4 |
| 6 | 2019 ncov.mp. | 4 |
| 7 | severe acute respiratory syndrome.mp. [mp=title, abstract, heading word, table of contents, key concepts, original title, tests & measures, mesh] | 292 |
| 8 | covid.mp. | 8 |
| 9 | (Wuhan adj3 virus).mp. | 0 |
| 10 | 1 or 2 or 3 or 4 or 5 or 6 or 7 or 8 or 9 | 387 |
| 11 | community services/ or exp mental health programs/ or exp mental health services/ or support groups/ | 69780 |
| 12 | exp Community Mental Health Services/ | 7657 |
| 13 | outreach programs/ or case management/ | 4205 |
| 14 | ((commun$ adj5 (mental health or model$ or pathway$1 or program$ or evaluat$ or intervention$ or implement$)) or camhs or cmht$1).ti,ab,id. | 64723 |
| 15 | exp Psychiatric Hospitals/ or exp Psychiatry/ or exp Psychiatric Hospitalization/ | 67702 |
| 16 | exp psychiatry/ | 50743 |
| 17 | (psychiatr$ adj3 (department$ or hospital$ or treatment$)).ti,ab,id. | 36316 |
| 18 | 11 or 12 or 13 or 14 or 15 or 16 or 17 | 203211 |
| 19 | (service$ or support$ or treatment$ or department$ or agenc$ or centre$ or center$ or clinic$).mp. [mp=title, abstract, heading word, table of contents, key concepts, original title, tests & measures, mesh] | 1933936 |
| 20 | exp mental disorders/ or exp mental health/ | 892485 |
| 21 | (mental$ adj2 (health or problem$ or disorder$ or ill$)).ti,ab,id. | 267147 |
| 22 | Mentally Ill Offenders/ | 3689 |
| 23 | Mentally ill/ | 0 |
| 24 | exp Cognitive Psychology/ or exp Clinical Psychology/ or exp Individual Psychology/ or psychology.mp. or exp Community Psychology/ | 334581 |
| 25 | exp Schizophrenia/ | 90125 |
| 26 | exp bipolar disorder/ | 30151 |
| 27 | (psychiatr$ or mental or SMI or psycho$ or schizo$ or manic or mania or bipolar or antipsycho*).mp. [mp=title, abstract, heading word, table of contents, key concepts, original title, tests & measures, mesh] | 1811448 |
| 28 | (depression or depressive or anxiety or phobi* or agoraphobi* or ADNOS or anxious or obsess* or compulsi* or panic or PTSD or "post traumatic stress" or "posttraumatic stress" or "stress disorder").ti,ab,id. | 467621 |
| 29 | "Depression (Emotion)"/ or Major Depression/ or Reactive Depression/ or Treatment Resistant Depression/ or anxiety disorders/ or generalized anxiety disorder/ or obsessive compulsive disorder/ or panic attack/ or panic disorder/ or phobias/ | 183717 |
| 30 | exp personality disorders/ | 26904 |
| 31 | ((personality or character) adj3 disorder$).mp. [mp=title, abstract, heading word, table of contents, key concepts, original title, tests & measures, mesh] | 45222 |
| 32 | (complex adj2 (needs or mental)).ti,ab,id. | 2990 |
| 33 | self-injurious behavior/ | 3976 |
| 34 | (self-harm or self-injury or self harm or self injury).ti,ab,id. | 8584 |
| 35 | Affective Disorders/ | 14061 |
| 36 | psychological disorder.mp. | 947 |
| 37 | (psychiatric adj2 (illness$ or disorder$)).mp. | 47170 |
| 38 | psychosocial disability.mp. | 174 |
| 39 | 20 or 21 or 22 or 23 or 24 or 25 or 26 or 27 or 28 or 29 or 30 or 31 or 32 or 33 or 34 or 35 or 36 or 37 or 38 | 2216966 |
| 40 | 19 and 39 | 1118242 |
| 41 | 10 and (18 or 40) | 95 |
| 42 | service user$.mp. | 6055 |
| 43 | ((expert$ or liv$) adj2 experience$).mp. [mp=title, abstract, heading word, table of contents, key concepts, original title, tests & measures, mesh] | 18458 |
| 44 | (consumer$1 or client$ or patient$ or outpatient? or out patient? or inpatient? or in patient?).mp. [mp=title, abstract, heading word, table of contents, key concepts, original title, tests & measures, mesh] | 948943 |
| 45 | 42 or 43 or 44 | 967056 |
| 46 | 39 and 45 | 648864 |
| 47 | 10 and 46 | 53 |
| 48 | (consultant$ or doctor? or employee$ or expert$ or facilitator$ or healthcare or instructor$ or leader$ or manager$ or mentor$ or nurs$ or personnel$ or specialist$ or staff$ or team$ or therapist$ or tutor$ or worker$ ir group$ or peer support$).mp. and 39 [mp=title, abstract, heading word, table of contents, key concepts, original title, tests & measures, mesh] | 349917 |
| 49 | exp Psychiatrists/ or exp Mental Health Personnel/ | 50965 |
| 50 | exp Clinical Psychologists/ | 2923 |
| 51 | exp Psychotherapists/ | 18101 |
| 52 | (psychiatrist$ or psychologist$ or psychotherapist$).mp. [mp=title, abstract, heading word, table of contents, key concepts, original title, tests & measures, mesh] | 145081 |
| 53 | (commun$ adj5 (service or hub$ or based or deliver$ or interact$ or led or maintenance or mediat$ or operated or provides or provider$ or run or setting$ or support or rehab$ or therap$ or service$ or treatment or management or assessment or assistance or care or day or week)).ti,ab,id. | 131810 |
| 54 | (network or outreach).mp. or ((specialist or day or whole) adj3 service).ti,ab,id. [mp=title, abstract, heading word, table of contents, key concepts, original title, tests & measures, mesh] | 102921 |
| 55 | 48 or 49 or 50 or 51 or 52 or 53 or 54 | 647830 |
| 56 | 10 and 55 | 100 |
| 57 | 41 or 47 or 56 | 145 |
| 58 | limit 57 to yr="2020 -Current" | 7 |

PUB MED

| [#16](https://www.ncbi.nlm.nih.gov/pubmed) | Search **((("2020/01/01"[Date - Publication] : "2020/04/30"[Date - Publication])) AND #15)** | [1](https://www.ncbi.nlm.nih.gov/pubmed/?cmd=HistorySearch&querykey=16)21 |
| --- | --- | --- |
| [#15](https://www.ncbi.nlm.nih.gov/pubmed) | Search **(#12 OR #13 OR #14)** | [195](https://www.ncbi.nlm.nih.gov/pubmed/?cmd=HistorySearch&querykey=15) |
| [#14](https://www.ncbi.nlm.nih.gov/pubmed) | Search **(#2 AND (#10 OR #11)) [COVID AND MENTAL HEALTH STAFF]** | [104](https://www.ncbi.nlm.nih.gov/pubmed/?cmd=HistorySearch&querykey=14) |
| [#13](https://www.ncbi.nlm.nih.gov/pubmed) | Search **(#2 AND (#8 AND #9)) [COVID AND MENTAL HEALTH PATIENTS]** | [32](https://www.ncbi.nlm.nih.gov/pubmed/?cmd=HistorySearch&querykey=13) |
| [#12](https://www.ncbi.nlm.nih.gov/pubmed) | Search **(#2 AND ((#8 AND #7) OR #3 OR #4 OR #5 OR #6)) [COVID AND MENTAL HEALTH SERVICES]** | [153](https://www.ncbi.nlm.nih.gov/pubmed/?cmd=HistorySearch&querykey=12) |
| [#11](https://www.ncbi.nlm.nih.gov/pubmed) | Search **((Psychiatrist* OR psychologist* or psychotherapist*))** | [43839](https://www.ncbi.nlm.nih.gov/pubmed/?cmd=HistorySearch&querykey=11) |
| [#10](https://www.ncbi.nlm.nih.gov/pubmed) | Search **((#8 AND (“doctor” OR “doctors” OR employee* OR facilitator* OR instructor* OR “leader” OR “leaders” OR manager* or mentor* OR nurs* OR personnel* OR specialist* OR staff* OR team* OR therapist* OR tutor* OR worker* OR “group” OR “peer support*” OR (commun* AND (provider* OR “support” OR supporter* OR “rehabilitator” OR therapist*)))))** | [506054](https://www.ncbi.nlm.nih.gov/pubmed/?cmd=HistorySearch&querykey=10) |
| [#9](https://www.ncbi.nlm.nih.gov/pubmed) | Search **(("service user*” OR “experts by experience*” OR “lived experience*” OR consumer* OR client* OR patient* OR outpatient* OR “out patient*” OR inpatient* OR “in patient*”))** | [3091679](https://www.ncbi.nlm.nih.gov/pubmed/?cmd=HistorySearch&querykey=9) |
| [#8](https://www.ncbi.nlm.nih.gov/pubmed) | Search **(((("mental health” OR “mental problem*” OR “mental disorder*” OR “mental illness*” OR “Depression” OR “Depressive disorder*” OR “Anxiety” OR "anxiety disorder*" OR "social* anxiety" OR phobi* OR agoraphobi* OR ADNOS OR "anxious” OR obsess* OR compulsi* OR “panic” OR PTSD or "post traumatic stress" OR "posttraumatic stress" OR "stress disorder*" OR Psychiatr* OR “SMI” OR psycho* OR schizo* OR “manic” OR “mania” OR “bipolar” OR “personality disorder*” OR (complex AND need*) OR self-harm OR self-injury OR “self harm” OR “self injury” OR “psychological disorder” OR (psychiatric AND (illness* OR disorder*)) OR “psychosocial disability”))) OR ((“Anxiety Disorders”[Mesh] OR “Bipolar Disorder”[Mesh] OR “Feeding and Eating Disorders”[Mesh] OR “Depressive Disorder”[Mesh] OR “Neurotic Disorders”[Mesh] OR “Personality Disorders”[Mesh] OR “Psychotic Disorders”[Mesh] OR “Schizophrenia”[Mesh] OR “Mental Disorders”[Mesh:NoExp] OR “Mental Health”[Mesh] OR "Mentally Ill Persons"[Mesh] OR "Self-Injurious Behavior"[Mesh] OR "Psychology, Clinical"[Mesh])))** | [1708581](https://www.ncbi.nlm.nih.gov/pubmed/?cmd=HistorySearch&querykey=8) |
| [#7](https://www.ncbi.nlm.nih.gov/pubmed) | Search **((Service*[Title/Abstract] OR support*[Title/Abstract] OR treatment*[Title/Abstract] OR department*[Title/Abstract] OR centre*[Title/Abstract] OR center*[Title/Abstract] OR clinic*[Title/Abstract]))** | [8950689](https://www.ncbi.nlm.nih.gov/pubmed/?cmd=HistorySearch&querykey=7) |
| [#6](https://www.ncbi.nlm.nih.gov/pubmed) | Search **((“Psychiatr* department*”[Title/Abstract] OR “psychiatr* hospital*”[Title/Abstract] OR “psychiatr* treatment*”[Title/Abstract]))** | [0](https://www.ncbi.nlm.nih.gov/pubmed/?cmd=HistorySearch&querykey=6) |
| [#5](https://www.ncbi.nlm.nih.gov/pubmed) | Search **(("Psychiatric Department, Hospital"[Mesh] OR "Hospitals, Psychiatric"[Mesh] OR "Psychiatry"[Mesh:NoExp]))** | [68268](https://www.ncbi.nlm.nih.gov/pubmed/?cmd=HistorySearch&querykey=5) |
| [#4](https://www.ncbi.nlm.nih.gov/pubmed) | Search **(("Mental Health Services"[Mesh:NoExp] OR "Community Mental Health Services" [Mesh:NoExp] OR "Community Mental Health Centers"[Mesh:NoExp] OR “Case Management”[Mesh:NoExp]))** | [62855](https://www.ncbi.nlm.nih.gov/pubmed/?cmd=HistorySearch&querykey=4) |
| [#3](https://www.ncbi.nlm.nih.gov/pubmed) | Search **((Outreach program*[Title/Abstract] OR “community mental health”[Title/Abstract] OR “community model*”[Title/Abstract] OR “community pathway*”[Title/Abstract] OR “community intervention*”[Title/Abstract] OR “camhs”[Title/Abstract]))** | [9982](https://www.ncbi.nlm.nih.gov/pubmed/?cmd=HistorySearch&querykey=3) |
| [#2](https://www.ncbi.nlm.nih.gov/pubmed) | Search **(("covid-2019" OR "covid 2019" OR "SARS cov 2" OR "SARS-cov-2" OR coronaviru* OR 2019-ncov))** | [16443](https://www.ncbi.nlm.nih.gov/pubmed/?cmd=HistorySearch&querykey=2) |

SSCI

| # 10 | [18](https://apps-webofknowledge-com.libproxy.ucl.ac.uk/summary.do?product=WOS&doc=1&qid=30&SID=F17rkBgQ2Xq5Q3bCtvG&search_mode=AdvancedSearch&update_back2search_link_param=yes) | #1 AND ((#4 AND #6) OR #7)  *Indexes=SCI-EXPANDED, SSCI, A&HCI, CPCI-S, CPCI-SSH, BKCI-S, BKCI-SSH, ESCI, CCR-EXPANDED, IC Timespan=Year to date* |
| --- | --- | --- |
| # 9 | [10](https://apps-webofknowledge-com.libproxy.ucl.ac.uk/summary.do?product=WOS&doc=1&qid=29&SID=F17rkBgQ2Xq5Q3bCtvG&search_mode=AdvancedSearch&update_back2search_link_param=yes) | #1 AND (#4 AND #5)  *Indexes=SCI-EXPANDED, SSCI, A&HCI, CPCI-S, CPCI-SSH, BKCI-S, BKCI-SSH, ESCI, CCR-EXPANDED, IC Timespan=Year to date* |
| # 8 | [23](https://apps-webofknowledge-com.libproxy.ucl.ac.uk/summary.do?product=WOS&doc=1&qid=28&SID=F17rkBgQ2Xq5Q3bCtvG&search_mode=AdvancedSearch&update_back2search_link_param=yes) | #1 AND ((#2 OR (#3 AND #4)))  *Indexes=SCI-EXPANDED, SSCI, A&HCI, CPCI-S, CPCI-SSH, BKCI-S, BKCI-SSH, ESCI, CCR-EXPANDED, IC Timespan=Year to date* |
| # 7 | [1,302](https://apps-webofknowledge-com.libproxy.ucl.ac.uk/summary.do?product=WOS&doc=1&qid=15&SID=F17rkBgQ2Xq5Q3bCtvG&search_mode=AdvancedSearch&update_back2search_link_param=yes) | ALL=(Psychiatrist* OR psychologist* or psychotherapist*)  *Indexes=SCI-EXPANDED, SSCI, A&HCI, CPCI-S, CPCI-SSH, BKCI-S, BKCI-SSH, ESCI, CCR-EXPANDED, IC Timespan=Year to date* |
| # 6 | [155,705](https://apps-webofknowledge-com.libproxy.ucl.ac.uk/summary.do?product=WOS&doc=1&qid=25&SID=F17rkBgQ2Xq5Q3bCtvG&search_mode=AdvancedSearch&update_back2search_link_param=yes) | ALL=((doctor* OR employee* OR facilitator* OR instructor* OR leader* OR mentor* OR nurs* OR personnel* OR specialist* OR staff* OR team* OR therapist* OR tutor* OR worker* OR “group” OR “peer support*” OR (commun* AND (provider* OR “support” OR supporter* OR “rehabilitator” OR therapist*))))  *Indexes=SCI-EXPANDED, SSCI, A&HCI, CPCI-S, CPCI-SSH, BKCI-S, BKCI-SSH, ESCI, CCR-EXPANDED, IC Timespan=Year to date* |
| # 5 | [127,209](https://apps-webofknowledge-com.libproxy.ucl.ac.uk/summary.do?product=WOS&doc=1&qid=24&SID=F17rkBgQ2Xq5Q3bCtvG&search_mode=AdvancedSearch&update_back2search_link_param=yes) | ALL=("service user*” OR “expert* by experience*” OR “lived experience*” OR patient* OR outpatient* OR “out patient*” OR inpatient* OR “in patient*”)  *Indexes=SCI-EXPANDED, SSCI, A&HCI, CPCI-S, CPCI-SSH, BKCI-S, BKCI-SSH, ESCI, CCR-EXPANDED, IC Timespan=Year to date* |
| # 4 | [61,250](https://apps-webofknowledge-com.libproxy.ucl.ac.uk/summary.do?product=WOS&doc=1&qid=6&SID=F17rkBgQ2Xq5Q3bCtvG&search_mode=AdvancedSearch&update_back2search_link_param=yes) | ALL=((("mental health” OR “mental problem*” OR “mental disorder*” OR “mental illness*” OR “Depression” OR “Depressive disorder*” OR “Anxiety” OR "anxiety disorder*" OR "social* anxiety" OR phobi* OR agoraphobi* OR ADNOS OR "anxious” OR obsess* OR compulsi* OR “panic” OR PTSD or "post traumatic stress" OR "posttraumatic stress" OR "stress disorder*" OR Psychiatr* OR “SMI” OR psycho* OR schizo* OR “manic” OR “mania” OR “bipolar” OR “personality disorder*” OR (complex AND need*) OR self-harm OR self-injury OR “self harm” OR “self injury” OR “psychological disorder” OR (psychiatric AND (illness* OR disorder*)) OR “psychosocial disability”)))  *Indexes=SCI-EXPANDED, SSCI, A&HCI, CPCI-S, CPCI-SSH, BKCI-S, BKCI-SSH, ESCI, CCR-EXPANDED, IC Timespan=Year to date* |
| # 3 | [528,653](https://apps-webofknowledge-com.libproxy.ucl.ac.uk/summary.do?product=WOS&doc=1&qid=5&SID=F17rkBgQ2Xq5Q3bCtvG&search_mode=AdvancedSearch&update_back2search_link_param=yes) | ALL=(Service* OR support* OR treatment* OR department* OR centre* OR center* OR clinic*)  *Indexes=SCI-EXPANDED, SSCI, A&HCI, CPCI-S, CPCI-SSH, BKCI-S, BKCI-SSH, ESCI, CCR-EXPANDED, IC Timespan=Year to date* |
| # 2 | [943](https://apps-webofknowledge-com.libproxy.ucl.ac.uk/summary.do?product=WOS&doc=1&qid=19&SID=F17rkBgQ2Xq5Q3bCtvG&search_mode=AdvancedSearch&update_back2search_link_param=yes) | ALL=(Outreach program* OR “community mental health” OR “community model*” OR “community pathway*” OR “community intervention*” OR “camhs” OR “Psychiatr* department*” OR “psychiatr* hospital*” OR “psychiatr* treatment*”)  *Indexes=SCI-EXPANDED, SSCI, A&HCI, CPCI-S, CPCI-SSH, BKCI-S, BKCI-SSH, ESCI, CCR-EXPANDED, IC Timespan=Year to date* |
| # 1 | [1,112](https://apps-webofknowledge-com.libproxy.ucl.ac.uk/summary.do?product=WOS&doc=1&qid=2&SID=F17rkBgQ2Xq5Q3bCtvG&search_mode=AdvancedSearch&update_back2search_link_param=yes) | ALL=("covid-2019" OR "covid 2019" OR "SARS cov 2" OR "SARS-cov-2" OR coronaviru* OR 2019-ncov)  *Indexes=SCI-EXPANDED, SSCI, A&HCI, CPCI-S, CPCI-SSH, BKCI-S, BKCI-SSH, ESCI, CCR-EXPANDED, IC Timespan=Year to date* |

CINAHL

| S14 | S8 OR S10 OR S12 (LIMIT TO PUBLICATION 2020) | (57) |
| --- | --- | --- |
| S13 | S8 OR S10 OR S12 | (518) |
| S12 | S11 AND (S1 OR S2) | (344) |
| S11 | (MH "Psychologists") OR (MH "Psychotherapists+")OR (MH "Psychiatrists") OR psychologist* OR psychiatrist* OR psychotherapist* OR (S6 AND (doctor* OR employee* OR facilitator* OR instructor* OR leader* OR manager* OR mentor* OR nurs* OR personnel* OR specialist* OR staff* OR team* OR therapist* OR tutor* OR worker* OR group* or "peer support*" OR (commun* AND (provider* OR support* or rehabilitat*)))) | (505,549) |
| S10 | S9 AND (S1 OR S2) | (218) |
| S9 | ((MH "Inpatients") OR (MH "Patients") OR (MH "Outpatients") OR (MH "Psychiatric Patients") OR "service user*" OR "expert* by experience*" OR ((lived or living) N1 experience*) OR consumer* OR client* OR patient* OR outpatient* OR out patient* OR inpatient* OR in patient*) AND S6 | (426,780) |
| S8 | (S7 OR S4 OR S3) AND (S1 OR S2) | (371) |
| S7 | S5 AND S6 | (630,176) |
| S6 | (MH "Adjustment Disorders") OR (MH "Mental Disorders") OR (MH "Mental Disorders, Chronic") OR (MH "Social Anxiety Disorders") OR (MH "Personality Disorders") OR (MH "Antisocial Personality Disorder") OR (MH "Avoidant Personality Disorder") OR (MH "Borderline Personality Disorder") OR (MH "Compulsive Personality Disorder") OR (MH "Dependent Personality Disorder") OR (MH "Affective Disorders, Psychotic") OR (MH "Psychotic Disorders") OR (MH "Paranoid Disorders") OR (MH "Schizoaffective Disorder") OR (MH "Schizophrenia") OR (MH "Psychiatric Emergencies") OR (MH "Psychological Trauma") OR (MH "Depression, Reactive") OR (MH "Depression") OR (MH "Bipolar Disorder") OR (MH "Anxiety") OR (MH "Social Anxiety Disorders") OR (MH "Anxiety Disorders") OR (MH "Generalized Anxiety Disorder") OR (MH "Stress Disorders, Post-Traumatic") OR (MH "Eating Disorders") OR (MH "Mental Health") OR (MH "Mentally Ill Offenders") OR (MH "Involuntary Commitment") OR (MH "Psychology, Clinical") OR (MH "Self-Injurious Behaviour") OR (MH "Injuries, Self-Inflicted") OR "mental health” OR “mental problem*” OR “mental disorder*” OR “mental illness*” OR “Depression” OR “Depressive disorder*” OR “Anxiety” OR "anxiety disorder*" OR "social* anxiety" OR phobi* OR agoraphobi* OR ADNOS OR "anxious” OR obsess* OR compulsi* OR “panic” OR PTSD or "post traumatic stress" OR "posttraumatic stress" OR "stress disorder*" OR Psychiatr* OR “SMI” OR psycho* OR schizo* OR “manic” OR “mania” OR “bipolar” OR “personality disorder*” OR (complex AND need*) OR self-harm OR self-injury OR “self harm” OR “self injury” OR “psychological disorder” OR (psychiatric AND (illness* OR disorder*)) OR “psychosocial disability” | (1,125,590) |
| S5 | service* or support* or treatment* or department* or agenc* or centre* or center* or clinic* | (3,066,747) |
| S4 | ((commun* N5 (mental health or model* or pathway* or program* or evaluat* or intervention* or implement*)) or camhs or cmht*)) OR psychiatr* N3 (department* or hospital* or treatment*)) or (mental health N3 (program* or support or service*) | (136,249) |
| S3 | (MH "Community Mental Health Services+") OR (MH "Mental Health Organizations+") OR (MH "Hospitals, Psychiatric") OR (MH "National Institute of Mental Health (U.S.)") OR (MH "Psychotherapy, Group+") OR (MH "Support Groups") OR (MH "Drug Rehabilitation Programs") OR (MH "Hospital Programs") OR (MH "Substance Use Rehabilitation Programs") OR (MH "Alcohol Rehabilitation Programs") OR (MH "Case Management") OR (MH "Multidisciplinary Care Team") OR (MH "Hospitals, Psychiatric") OR (MH "Psychiatry+")  [...](javascript:showHistoryTerm('ctl00_ctl00_FindField_FindField_historyControl_HistoryRepeater_ctl25_ellipsis',true)) | (157,423) |
| S2 | (cov* N4 2019) or "covid-2019" or "SARS cov 2" or "SARS-cov-2" or "2019-ncov" or "2019 ncov" or "severe acute respiratory syndrome" or "covid" or (wuhan N3 virus) or coronaviru* | (4,783) |
| S1 | (MH "Coronavirus+") OR (MH "Coronavirus Infections+") OR (MH "SARS Virus") | (3,313) |

**Other sources**

##### Blogs*, online commentaries, and grey literature (published online from January 2020 until April 2020)

- Publicly available first person accounts*, commentaries, articles, reports, narratives and media (including videos) published online

*Note on consent and the use of blogs for research: Blogs in the public domain are considered to be ‘grey literature’ (Thompson, 2017). We will assume that first person accounts published on organisational websites, in journals or on other such sources are publicly available. For any other blogs retrieved from searches, such as personal blogs, permission will be sought from the author if it appears to relate to a code that is not already well-covered, and the item included if permission is given.

###### **Sources.**

1. **A targeted search using google advanced search, including (+Blog specific search) , blogsearchengine.com, and metasearch engine DevonAGENT express** will be conducted to identify relevant information using a combination of the following terms:

“covid” or “coronavirus” AND (“mental health care” or “mental health service” or “mental health problem” or “mental illness” or “psychiatric” or “depression” or “anxiety” or “eating disorder” or “bipolar” or “schizophrenia” or “psychosis” or “panic” or “obsessive compulsive disorder” or “ocd” or “self harm” or “suicide” or “personality disorder”).

The first 500 results of each search will be scanned for inclusion.

**In addition, websites relevant to mental health will be searched**

These websites will be searched for the following terms:

“coronavirus” or “covid”.

The first 100 results will be scanned for inclusion for each search.

In addition to searches, websites will be scanned for recent content related to COVID-19, and relevant twitter feeds searched for relevant content

1. **The following NHS and professional organisation websites will also be searched:** NHS England; NHS Confederation, Public Health England, Royal College of Nursing, Royal College of Psychiatrists, Royal College of Occupational Therapists, British Association of Social Workers, Association of Directors of Adult Social Care Services, The Health Care Professionals Council, Nursing and Midwifery Council, British Psychological Society, British Association for Cognitive and Behavioural Psychotherapies, Unison, Unite the Union (Mental Health Nurses Association)

These websites will be searched for the following terms:

(“covid” OR “coronavirus”) AND (“mental health care” OR “mental health service” OR “mental health problem” OR “mental illness” OR “psychiatric” OR “depression” OR “anxiety” OR “eating disorder” OR “bipolar” OR “schizophrenia” OR “psychosis” OR“panic” OR “obsessive compulsive disorder” OR “ocd” OR “self harm” OR “suicide” OR “personality disorder” ).

The first 100 results will be scanned for inclusion for each search.

In addition to searches, websites will also be scanned for recent content related to COVID-19, and relevant twitter feeds searched for relevant content

**Additional targeted searches**

- We will conduct searches for non-English language articles (French, Spanish, Italian, German, Chinese):
  - Google and Google Scholar searches for translated terms (first 100 results scanned for inclusion)
  - Examination of websites of major national charities and professional bodies
- Searches for international guidance relevant to COVID-19 will be conducted in google

##### Additional source identification and search methods

- Contact with experts in the field.
- Search for tweets with links to articles
- A priori identified topics not picked up in original searches will be targeted in an additional google search

# **Section 3 – Online Data Collection Form**

Rapid synthesis of Impact of Covid-19 on Mental Health Care

*Guidance*

We would like you to use the headings and sub-headings below to summarise the main content of each article. Please try to include the main points made. If there is numerical data, please include it. If there is very extensive material that you think is very interesting, please include a link and details of where to find this material. You may copy and paste material from the article if that is the easiest way to create a quick summary, but please use inverted commas to make it clear where you have done this.

Most articles are relevant to only a few sub-headings, so don’t worry if you use only a few, or even only one. Just leave gaps elsewhere. If you find any particularly good quotes or text, please do include these too (and indicate that it is copied/pasted text). All the boxes will expand as much as needed.

There may well be interesting material that is relevant to our main questions but doesn’t fit with any of the sub-headings. If so, please summarise this in one of the OTHER boxes at the end of each section. Don’t worry too much about whether things are in the right section – we can easily move things if we need to

Source details:_________________

Source title: _________________

Author(s): _________________

Language the article is written in:

- English
- Chinese
- French
- German
- Italian
- Spanish

Link to article (URL): _________________

Date accessed (DD/MM/YYYY): _________________

Source type:

- Journal article
- Report
- Blog (published)
- Organisational website
- Media article
- Other (please specify)

Country or countries being written about: _________________

Setting: Is the article about impact on mental health care in general or on a particular service setting? If a particular service setting (acute inpatient, crisis service, residential service etc.), please describe:_________________

Client group /type of mental health problem: Is it about impact on people with mental health problems in general or about a specific type of problem, or a specific population (e.g. a diagnostic or demographic group?): _________________

Author type (select all that apply):

- Scientist
- Clinician
- Journalist
- Policy maker
- Person with relevant lived experience
- Other (please describe)

Article summariser: _________________

Email address: _________________

**1. Direct impacts on mental health service users and carers**

Does the paper describe any impacts of the pandemic and its consequences on the needs and experiences of people with mental health problems?

(We are primarily interested in the experiences of people who already had mental health problems prior to the pandemic, not in new mental health problems arising from the pandemic. Included here may be both descriptions of changes and impacts experienced by individuals, and aggregated evidence on changes in needs and difficulties etc. among people with mental health problems).

- Yes
- No

If Yes, please summarise under the following sub-headings. Put anything that relates to the topic and that doesn't obviously fit in the other boxes in the OTHER box below:

1) Impacts on the general well-being of people with mental health problems, and on symptoms and signs of particular conditions (e.g. OCD, eating disorders, depression, anxiety conditions, self harm and suicidal behaviour, psychosis, bipolar, people with a "personality disorder" diagnosis)

2) Impacts on health-relevant behaviours such as eating patterns, exercise, smoking, alcohol and drug use, gambling

3) Effects of greater social isolation and loneliness, and of restriction of activities and contacts

4) Experiences of having COVID-19 infection among people with mental health problems

5) Risk of getting COVID-19 and its impact, including health anxiety regarding COVID-19 among people who already had mental health problems, and concerns or evidence about people with mental health problems being at greater risk of infection/severe illness

6) Difficulties directly related to infection control guidance (e.g. related to social distancing, self-isolation and shielding), including difficulties in following this while living with mental health problems and legal repercussions of such difficulties. (But use the box above to summarise material on loneliness and social isolation)

7) Impacts of any new challenges in getting access to necessary services during the pandemic, including mental and physical healthcare, primary and social care. Concerns about equitable access to services, especially physical healthcare, during the pandemic period.

8) Practical and social difficulties in getting access to food, money, adequate accommodation. Effects of the pandemic on social inequalities already experienced by people with mental health problems

9) Exacerbation of safeguarding risks, such as domestic violence and risk to children, effects on people with mental health problems of these

10) Effects on families, friends and carers

11) Any positive experiences of life during the pandemic (e.g. feeling less excluded or less under pressure, awareness of resilience in adversity, renewal of connections

OTHER. Please write here about any impacts on people with mental health problems and on carers that you have not readily been able to fit under any of the above headings.

**2. Self-help and informal help strategies**

Does this source describe strategies that people use themselves to try and cope with any of the other above difficulties? Include here self-management strategies, use of online tools and apps (apart from those that are part of mental health treatment), other informal strategies such as peer support or help from neighbours or local volunteers.

(In general don’t include generic coping advice on official websites, but include strategies that people describe experience of trying to use.)

- Yes
- No

If Yes, please summarise under the following sub-headings. Put anything that relates to the topic and that doesn't obviously fit in the other boxes in the OTHER box below:

1) Individual self-management strategies that people develop, how these have worked

2) Use of self-management and virtual therapy tools, including apps and online interventions and of websites, online support groups and helplines. Include here tools that people have found themselves and/or that are not part of what mental health and primary care services provide. There is space further on for describing use of new digital tools in mental health services. (If you aren't sure exactly where to code something, don't worry too much and please just make sure you put it in somewhere).

3) Peer support and mutual aid among people with mental health problems and carers

4) Help from neighbours and wider local communities

5) Coping strategies employed by carers/family members

OTHER: Is there any material about self-management and the strategies that people find themselves to manage current challenges? Please put anything that doesn't fit into the above headings here. Please note that there are headings below about the mental health service response.

**3. Impact on needs for and use of mental health care**

Does this source describe changes in use of mental health care, in demand for services, and in types of mental health care being sought?

We are interested both in aggregate data on increased or decreased use of or demand for mental health treatments and services (e.g. admission rates, referral rates, calls to crisis lines, medication prescriptions), and in more subjective reports on changes in use and demands.

- Yes
- No

If Yes, please summarise under the following sub-headings. Put anything that relates to the topic and that doesn't obviously fit in the other boxes in the OTHER box below:

1) Any data on whether mental health service use or demands for mental health treatments and services (e.g. admission rates, referral rates, calls to crisis lines, medication prescriptions) have changed during the pandemic period (give a link if the data is too extensive to summarise here)

2) Any impressions in how use of and demand for have changed during the pandemic period e.g. clinicians writing about how they think patterns of use have changed.

3) Any new type of mental health problem that mental health clinicians report seeing and believe are directly related to the pandemic

OTHER: Use this box for anything that doesn't seem to fit well in the sub-headings above.  Please include here anything that relates in changes in demand or in the needs that people in mental health services are encountering. Note that there are headings below for specific challenges faced in mental health services, like infection control and workforce problems, and for ways in which mental health services have adapted.

**4. Challenges faced in mental health services**

Does this source describe challenges faced by providers of mental health care during the pandemic (apart from those directly arising from service user needs already described)?

- Yes
- No

If Yes, please summarise under the following sub-headings. Put anything that relates to the topic and that doesn't obviously fit in the other boxes in the OTHER box below:

1) Infection control challenges in mental health settings and how they are being managed

2) Difficulties for services and clinicians in delivering a sufficient quality services and necessary treatments effectively in current circumstances

3) Workforce challenges e.g. due to staff sickness or re-deployment, or low morale (we are not including material that primarily focuses on staff well-being, but include if part of a wider discussion of challenges for mental health care)

4) Challenges in maintaining professional values and human rights (e.g due to difficulties in implementing usual deprivation of liberty and involuntary admission processes or safeguarding processes, difficulties ensuring equitable treatment of service users)

5) Future challenges and persisting problems anticipated by authors

OTHER: Please use this box for any challenges being encountered in delivering mental health services that do not readily fit under the sub-headings above.

**5. Changes in availability and accessibility of mental health care, innovations and adaptations**

Does this source describe any change in availability and  accessibility of mental health care since the pandemic began, or any adaptation or innovation in mental health care?

- Yes
- No

If Yes, please summarise under the following sub-headings. Put anything that relates to the topic and that doesn't obviously fit in the other boxes in the OTHER box below:

1) Changes in what is available from mental health services (including social care and voluntary sector services that provide mental health care) and the effects of these, including reports of variations between areas in what is available

2) Innovations made in mental health care to meet needs in the current situation, experiences and effects of these

3) Greater adoption of digital and remote tools to allow mental health care needs to be met in current situation, adoption of new methods for clinical contacts, digital innovations, experiences and effects of these

4) Support or services that have not changed, or that mental health providers have felt it important to maintain/keep the same

5) Guidelines regarding how services should be delivered, notes on their implementation (do not attempt to go into great detail with such recommendations, but give us a link to where they may be found)

6) Discussion of how the pandemic and associated changes may affect future mental health care

OTHER: Please summarise here any material about how mental health care has changed or about innovations that does not fit well with any of the above headings.

**6. Other**

Write anything here that might be relevant to the research questions that has not already been covered. The research questions are:

1) What is known from the existing literature about the current and potential impacts of COVID-19 on mental health services and the people that use and work in them?

2) What efforts/innovations to combat impacts of COVID-19 on mental health services are described in the literature and how useful are they?

3) Please write any further reflections you have about the style and content of the article that you think it may be important for those doing the final coding to be aware of.
